# Multiomic profiling of new-onset kidney function decline: insights from the STANISLAS study cohort with a 20-year follow-up

**DOI:** 10.1093/ckj/sfae224

**Published:** 2024-07-18

**Authors:** Vincent Dupont, Constance Xhaard, Isabelle Behm-Ansmant, Emmanuel Bresso, Quentin Thuillier, Christiane Branlant, Marilucy Lopez-Sublet, Jean-François Deleuze, Faiez Zannad, Nicolas Girerd, Patrick Rossignol

**Affiliations:** Department of Nephrology, University hospital of Reims, Reims, France; FCRIN INI-CRCT (Cardiovascular and Renal Clinical Trialists ); CNRS UMR 7369, Université de Reims Champagne-Ardenne, Reims, France; FCRIN INI-CRCT (Cardiovascular and Renal Clinical Trialists ); Université de Lorraine, Centre d'Investigations Cliniques- Plurithématique 14-33, Inserm U1116, CHRU Nancy, France; Université de Lorraine, CNRS, UMR 7365, IMoPA, Nancy, France; FCRIN INI-CRCT (Cardiovascular and Renal Clinical Trialists ); Université de Lorraine, Centre d'Investigations Cliniques- Plurithématique 14-33, Inserm U1116, CHRU Nancy, France; Université de Lorraine, CNRS, UMR 7365, IMoPA, Nancy, France; Université de Lorraine, CNRS, UMR 7365, IMoPA, Nancy, France; FCRIN INI-CRCT (Cardiovascular and Renal Clinical Trialists ); AP-HP, Hopital Avicenne, Centre d'Excellence Europeen en Hypertension Arterielle, Service de Medecine Interne, INSERM UMR 942 MASCOT, Paris 13-Universite Paris Nord, Bobigny, France; Centre National de Recherche en Génomique Humaine, Institut François Jacob, CEA, Université Paris-Saclay, Evry, France; FCRIN INI-CRCT (Cardiovascular and Renal Clinical Trialists ); Université de Lorraine, Centre d'Investigations Cliniques- Plurithématique 14-33, Inserm U1116, CHRU Nancy, France; FCRIN INI-CRCT (Cardiovascular and Renal Clinical Trialists ); Université de Lorraine, Centre d'Investigations Cliniques- Plurithématique 14-33, Inserm U1116, CHRU Nancy, France; FCRIN INI-CRCT (Cardiovascular and Renal Clinical Trialists ); Medicine and Nephrology-dialysis departments, Princess Grace Hospital, and Monaco Private Hemodialysis Centre, Monaco, Monaco

**Keywords:** glomerular filtration rate, proteomic, transcriptomic

## Abstract

**Background:**

Identifying the biomarkers associated with new-onset glomerular filtration rate (GFR) decrease in an initially healthy population could offer a better understanding of kidney function decline and help improving patient management.

**Methods:**

Here we described the proteomic and transcriptomic footprints associated with new-onset kidney function decline in an initially healthy and well-characterized population with a 20-year follow-up. This study was based on 1087 individuals from the familial longitudinal Suivi Temporaire Annuel Non-Invasif de la Santé des Lorrains Assurés Sociaux (STANISLAS) cohort who attended both visit 1 (from 1993 to 1995) and visit 4 (from 2011 to 2016). New-onset kidney function decline was approached both in quantitative (GFR slope for each individual) and qualitative (defined as a decrease in GFR of >15 ml/min/1.7 m^2^) ways. We analysed associations of 445 proteins measured both at visit 1 and visit 4 using Olink Proseek^®^ panels and 119 765 genes expressions measured at visit 4 with GFR decline. Associations were assessed using multivariable models. The Bonferroni correction was applied.

**Results:**

We found several proteins (including PLC, placental growth factor (PGF), members of the tumour necrosis factor receptor superfamily), genes (including *CCL18, SESN3*), and a newly discovered miRNA—mRNA pair (MIR1205–DNAJC6) to be independently associated with new-onset kidney function decline. Complex network analysis highlighted both extracellular matrix and cardiovascular remodelling (since visit 1) as well as inflammation (at visit 4) as key features of early GFR decrease.

**Conclusions:**

These findings lay the foundation to further assess whether the proteins and genes herein identified may represent potential biomarkers or therapeutic targets to prevent renal function impairment.

## Graphical Abstract

**Graphical Abstract:**
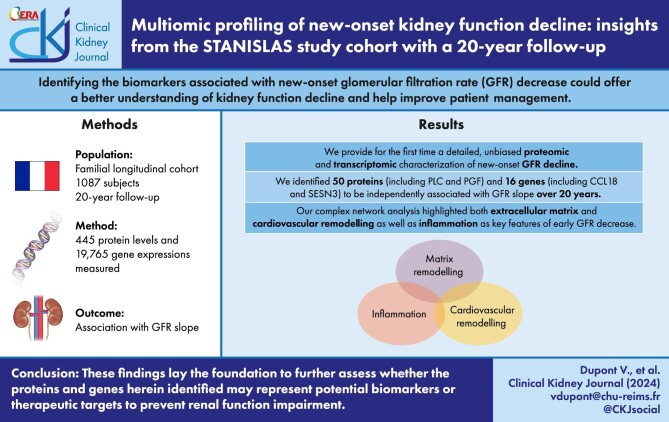

KEY LEARNING POINTS **What was known**:

- Multiomic analysis of large cohorts has already allowed to generate multidimensional datasets that improved knowledge on the pathophysiology of multiple diseases.
- Identifying the biomarkers associated with new-onset glomerular filtration rate (GFR) decrease could offer a better understanding of kidney function decline and help improve patient management.

**This study adds**:

- Here, we report for the first time the associations between thousands of proteins levels and genes expressions with new-onset kidney function decline over a 20-year period in a large, initially healthy population without comorbidities and using an unbiased multiomic approach.
- The expressions of 50/445 proteins and 16/119 765 genes were independently associated with eGFR slope over 20 years.
- Our complex network analysis highlighted both extracellular matrix and cardiovascular remodelling as well as inflammation as key features of early GFR decrease.

**Potential impact**:

- Our findings lay the foundation to further assess whether the proteins and genes herein identified may represent potential biomarkers or therapeutic targets of interest to prevent renal function impairment.

## INTRODUCTION

Chronic kidney disease (CKD) represents a global healthcare burden affecting 10% of the worldwide population and is projected to become the fifth leading cause of death by 2040 [1, 2]. Whatever the cause, CKD natural history may lead to end-stage renal disease. Its associated morbidity and mortality, mostly from cardiovascular diseases, increase along with glomerular filtration rate (GFR) decline [3]. Therefore, early diagnosis and management of CKD may offer the potential to substantially reduce the burden from renal function impairment [3, 4]. Hence, identifying potential biomarkers and pathophysiological mechanisms associated with new-onset renal disease could be of major importance, not only for a better understanding of the disease but also for improving the current prevention strategies and treatments [5].

In recent years, high-throughput technologies have considerably revolutionized medical research [6]. Multiomic analysis of large cohorts has already allowed to generate new and multidimensional datasets that improved knowledge on multiple diseases such as cancer, diabetes, and cardiovascular diseases [7–9]. Nowadays, both transcriptomic and proteomic measurements are easily accessible and may represent a promising alternative to better assess early renal function impairment as well [10, 11].

We aimed to describe the transcriptomic and proteomic factors associated with new-onset kidney function decline in an initially healthy, well-characterized population with a 20-year follow-up.

## MATERIALS AND METHODS

### Study cohort

The STANISLAS (Suivi Temporaire Annuel Non-Invasif de la Santé des Lorrains Assurés Sociaux) cohort is a single-centre familial prospective cohort of 1006 healthy families (4295 participants) from the Nancy region of France recruited from 1993 to 1995 for visit 1 (V1), then followed each for 5 to 10 years [12]. Visit 4 (V4) took place from 2011 to 2016 and included 1705 participants. Out of 4295 participants, 111/ (2.6%) died between V1 and V4 [12]. Participants and nonparticipants at V4 had similar baseline characteristics [13].

Beyond the clinical, imaging, and routine laboratorial measurements, circulating levels of multiple proteins were assessed at V1 and V4. In addition, a genome-wide association study (GWAS) and a global transcriptome analysis were performed for only the V4 participants. This design allows the assessment of the clinical factors, genetic variants, and transcriptomic and proteomic signatures associated with new-onset pathologies such as early kidney function decline. In the present analysis, we included 1087 participants who attended both V1 and V4 and were at least 18 years old at V1.

The research protocol was conducted according to the Declaration of Helsinki and approved by the local ethics committee (Comité de Protection des Personnes Est III, Nancy, France). All study participants gave written informed consent to participate.

### New-onset kidney function decline definition

GFR decline is causally associated with renal function impairment [14]. Recent data support the use of GFR slope as a primary endpoint for clinical trials focusing on CKD progression [15]. Thus, we chose to approach new-onset kidney function decline in our cohort using two ways: (i) as a quantitative variable, i.e. the estimated GFR (eGFR) slope between V1 and V4 for each individual, and (ii) as a qualitative variable, i.e. a decrease in eGFR (ΔeGFR) of >15 ml/min/1.7 m^2^ between V1 and V4 based on the CKD-EPI formula and using plasma creatinine levels [16]. Creatinine measurements were performed using the Jaffe method at V1 and enzymatic method at V4.

### Gene expression and circulating proteins

Baseline (V1) and follow-up (V4) plasma samples were analysed by the TATAA-biocentre using the Olink Proseek^®^ Multiplex cardiovascular II, III, cardiometabolic, organ-damage and inflammatory panels using a proximity extension assay technology [17]. A total of 445 proteins were available for statistical analysis.

Blood DNA was extracted using Gentra Puregene Blood Kit (Qiagen, Germany®) and stored at −20°C only for V4 participants. Genotyping was conducted at the Centre National de Recherche en Génomique Humaine (Evry, France) using the Illumina Global Screening and Exome Arrays [18].

Genes expressions were assessed on a subgroup of 887 individuals having available transcriptomic data. Whole blood RNAs were extracted from PAXgene Blood RNA Tubes (Qiagen, Germany) using MagMAX Isolation Kit (Life Technologies, France) on a KingFisher Duo Prime automated purification system (ThermoFisher Scientific, France). Extracted RNAs were quantified using a Nanodrop spectrophotometer, and their quality was assessed using the RNA ScreenTape system (Agilent, France). Transcriptome analysis was conducted at the Ingénierie Moléculaire et Physiopathologie Articulaire (Vandoeuvre-les-Nancy, France) using Clariom D^®^ assays (Affymetrix, ThermoFisher Scientific, France) [19]. Gene expressions intensities were normalized using ‘Limma’ [20] (v.3.44.3) implemented in the Transcription Analysis Console Software v.4.0.2 by SST-RMA and then extracted for each individual as normalized intensity values (as log_2_) obtained from Clariom D^®^ microarrays.

### Statistical and bioinformatical analysis

For the baseline and follow-up clinical characteristics, continuous variables are expressed as means and standard deviations and compared using Student's *t*‐test or Wilcoxon nonparametric tests. Categorical variables are presented as frequencies and percentages and compared using the Chi-squared test.

The heritability of a phenotype is defined as the proportion of its total variance explained by additive genetic factors. Heritability is optimally estimated in family-based sample populations. Here, we took advantage from the familial structure of our cohort to approach the heritability of eGFR decline. To this aim, we considered both our study population (*n* = 1087) and their children aged <18 years at V1 (total *n* = 1420). We used a linear mixed model that simultaneously included additive genetic effects across the genome, common environment effects shared by the nuclear family and fixed effects. The additive genetic effects were assessed using the Genetic Relatedness Matrix [21]. Estimations were performed using R (v.3.4.1.) and were implemented in the ‘gaston’ R package (v.1.5.6).

Associations between each of the proteins/genes and eGFR slope between V1 and V4 (ml/min/1.7 m^2^) were assessed using multivariable linear regression models. To strengthen our approach, we also tested the associations between each of the proteins and genes and ΔeGFR > 15 ml/min/1.7 m^2^ using multivariable logistic regression models. For both approaches, only genes that were associated with eGFR decline with a false discovery rate <10^−4^ in univariate analysis were included in multivariate analysis.

The following variables were included in multivariable analysis models: age, sex, body mass index (BMI), current smoker, and eGFR [22]. Baseline eGFR was accounted for in multivariate analysis as a major determinant of eGFR slope as previously suggested [23]. To account for multiplicity, we systematically applied the Bonferroni correction to the *P* values obtained in multivariate analysis [24].

To assess the potential ability of each of the proteins and genes to early identify kidney function decline, we generated receiver operating characteristic (ROC) curves (with 95% confidence interval) for the top ones associated with ΔeGFR ≥ 15 ml/min/1.7 m^2^.

A complex network analysis was performed using the Fight Heart-Failure Graph Knowledge Box (FHF-GKB) [25]. Proteins were imported from UniProt [26]. Pathways and protein-pathway relationships were retrieved from REACTOME [27]. Resulting networks were displayed by Cytoscape (v.3.9.1).

## RESULTS

### Characteristics of the study population

**Figure 1:**
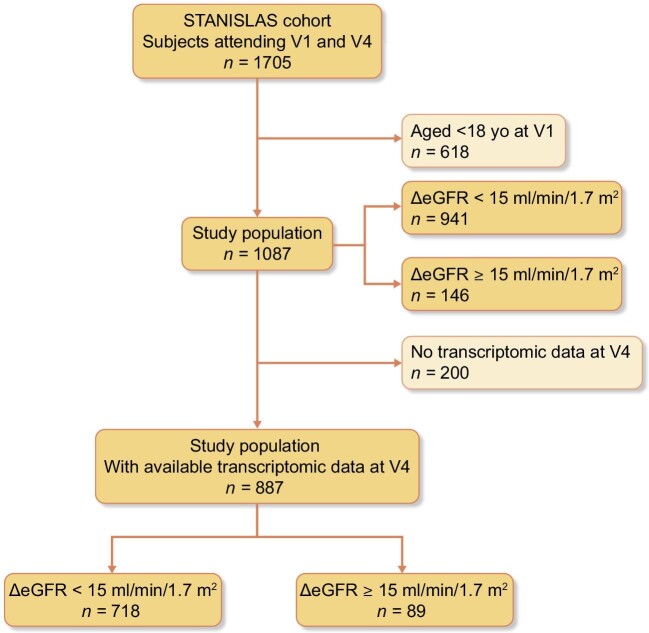
Flow chart of the study.

The flow chart of the study is depicted in Fig. 1. The characteristics of the 1087 included individuals, both at V1 and V4, are summarized in Table 1. Subjects were relatively young (40 ± 8 years) and showed no comorbidities at baseline. Overall, eGFR remained stable within the 20-year follow-up in the whole population (−1.9 ± 12.2 ml/min/1.7 m^2^). Between V1 and V4, 146/1087 (13.4%) subjects experienced ΔeGFR ≥ 15 ml/min/1.7 m^2^, 75/1087(6.9%) experienced ΔeGFR ≥ 20 ml/min/1.7 m^2^, and 15/1087 (1.4%) experienced ΔeGFR ≥ 30 ml/min/1.7 m^2^ (Supplemental Fig. S1). V4 eGFR was <60 ml/min/1.7 m^2^ in 15/1087 individuals.

**Table 1:** Characteristics: of the study population and according to eGFR decline status.

	**Overall**	**ΔeGFR < 15 ml/min/1.7** **m^2^**	**ΔeGFR ≥ 15 ml/min/1.7** **m^2^**	
	**(*n* = 1087)**	**(*n* = 941)**	**(*n* = 146)**	***P* value**
**V1**				
Sex female	557(51.2)	490(52.1)	67(45.9)	.19
Age (y)	40 ± 8	39 ± 8	40 ± 8	.64
BMI (kg/m²)	24.1 ± 3.7	24.2 ± 3.7	23.9 ± 3.6	.43
Current smoker	236 (21.8)	203 (21.6)	33 (22.76)	.85
NSAID	14 (1.3)	11 (1.2)	3 (2.1)	.90
eGFR (ml/min/1.73 m²)	91.7 ± 13.6	90.5 ± 13.2	99.5 ± 13.3	<.001
**V4**				
Sex female	557 (51.2)	490(52.1)	67 (45.9)	.19
Age (y)	58 ± 8	58 ± 8	58 ± 8	.90
BMI (kg/m²)	26.7 ± 4.9	26.8 ± 4.9	26.4 ± 4.4	.37
Current smoker	155 (14.3)	136 (14.5)	19 (13.1)	.75
Diabetes	88 (8.1)	78 (8.3)	10 (6.9)	.67
Anti-diabetic medication	56 (5.4)	47 (5.0)	9 (6.2)	.69
Total cholesterol (g/l)	2.2 ± 0.4	2.2 ± 0.4	2.2 ± 0.4	.14
LDL (g/l)	1.4 ± 0.3	1.4 ± 0.3	1.4 ± 0.3	.19
HDL (g/l)	0.6 ± 0.1	0.6 ± 0.1	0.6 ± 0.1	.81
Urine ACR (mg/mmol)	1.7 ± 10.2	1.3 ± 4.6	4.3 ± 25.4	.16
Hypertension	322 (29.6)	273 (29.0)	49 (33.6)	.31
Diuretic	115 (10.1)	91 (9.7)	24 (16.4)	.02
RAAS blocker	75 (6.9)	63 (6.7)	12 (8.2)	.62
Calcium channel blocker	92 (8.5)	79 (8.4)	13 (8.9)	.96
Beta blocker	130 (11.2)	105 (11.2)	25 (17.1)	.05
Statin	204 (18.8)	169 (18.0)	35 (24.0)	.11
NSAID	36 (3.4)	32 (3.4)	4 (2.7)	.87
eGFR (ml/min/1.73 m²)	89.8 ± 12.6	91.7 ± 11.2	77.7 ± 14.8	<.001
ΔeGFR (ml/min/1.73 m^2^)	−1.9 ± 12.2	1.2 ± 9.7	-21.8 ± 6.3	<.001

Variables are expressed as number(percentage) or mean ± standard deviation. Multivariable logistic regression model includes the following variables: following variables at V1: age, sex, BMI, current smoker, and eGFR. LDL, low density lipoprotein; HDL, high density lipoprotein; ACR, albumin to creatinine ratio; RAAS, renin angiotensin aldosterone system. NSAID, non-steroidal anti-inflammatory drug.

Individuals who experienced ΔeGFR ≥ 15 ml/min/1.7 m^2^ had a significantly lower eGFR at V4 (77.7 ± 14.8 vs 91.7 ± 11.2 ml/min/1.7 m^2^, *P *< .001) despite a higher baseline eGFR (99.5 ± 13.3 vs 90.5 ± 13.2 ml/min/1.7 m^2^, *P *< .001). There was no demographic difference at baseline between the individuals who further experienced ΔeGFR ≥ 15 ml/min/1.7 m^2^ and those who did not (Table 1). At V4, individuals who experienced ΔeGFR ≥ 15 ml/min/1.7 m^2^ showed higher rates of antihypertensive drugs use (diuretics and beta-blockers) as well as an increasing trend in urine albumin to creatinine ratio (Table 1). There was no difference in terms of cardiovascular characteristics at V4 between the two groups (Supplemental Table S1).

### Heritability

First, we aimed to assess the heritability of new-onset kidney function decline using the genetic intrafamilial correlations calculated from the GWAS data of our study cohort. Our model allowed us to decompose the total variance of eGFR decline in three components: heritability, common environment effect, and residual effect (i.e. personal environment). We found moderate heritability (30.23%) for eGFR slope between V1 and V4. Common environment and residual effects accounted for 3.76% and 66.01%, respectively.

### Proteins/genes associated with eGFR slope

**Figure 2:**
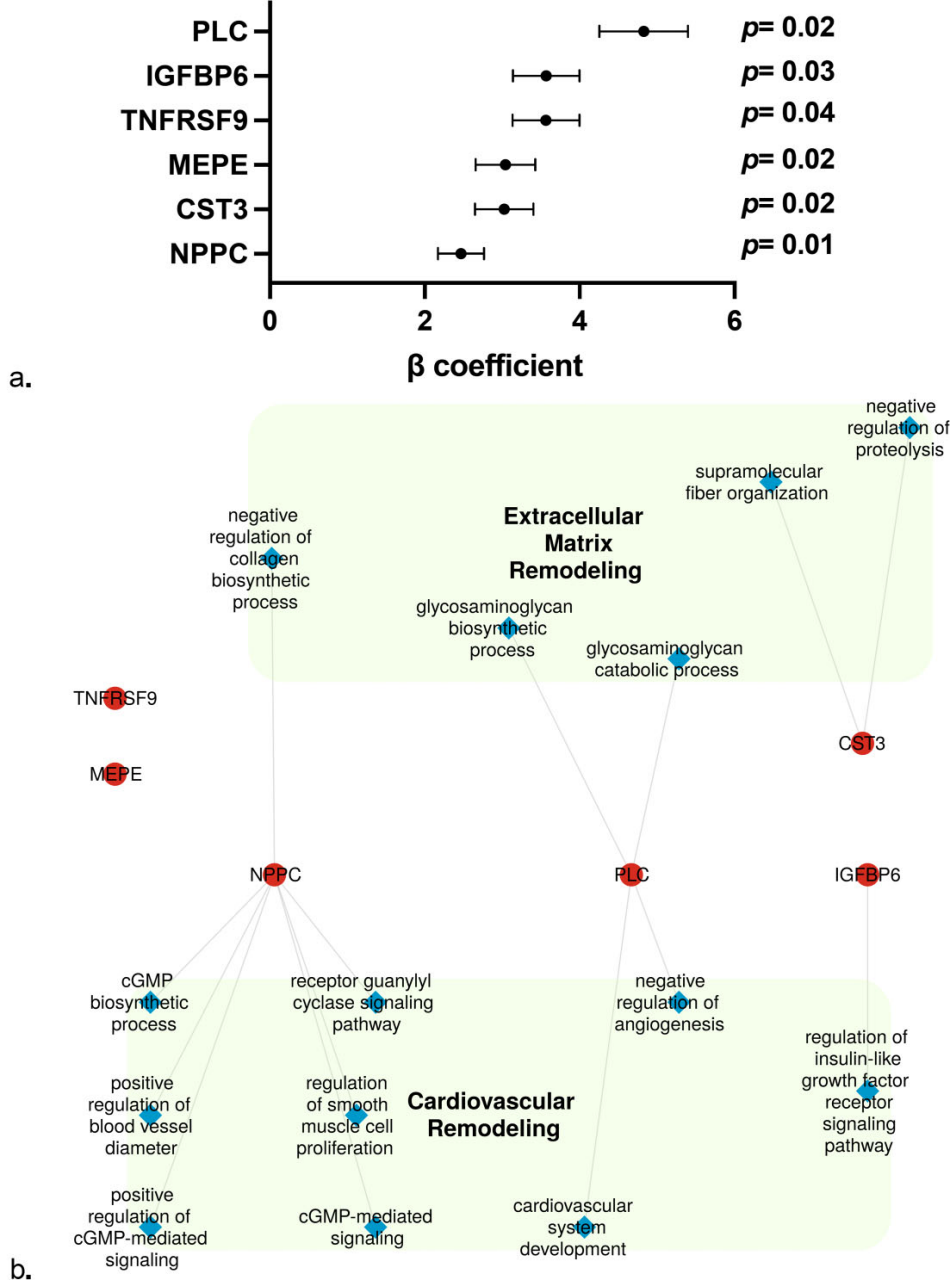
Associations between proteins levels measured at V1 and eGFR slope. (**a**) Forrest plot of proteins levels measured at V1 independently associated with eGFR slope. Multivariable linear regression model includes the following variables at V1: age, sex, BMI, current smoker, and eGFR. Dots represent β coefficient and standard error for each protein. P values (with Bonferroni correction) are indicated. (**b**) Network depiction of proteins co-expression modules that are positively correlated with eGFR slope at V1. Red dots represent proteins and blue diamonds represent biological process connecting the overrepresented proteins. NPPC, natriuretic peptide precursor C; IGFBP6, insulin-like growth factor binding protein-6; CTS3, cystatin C.

**Figure 3:**
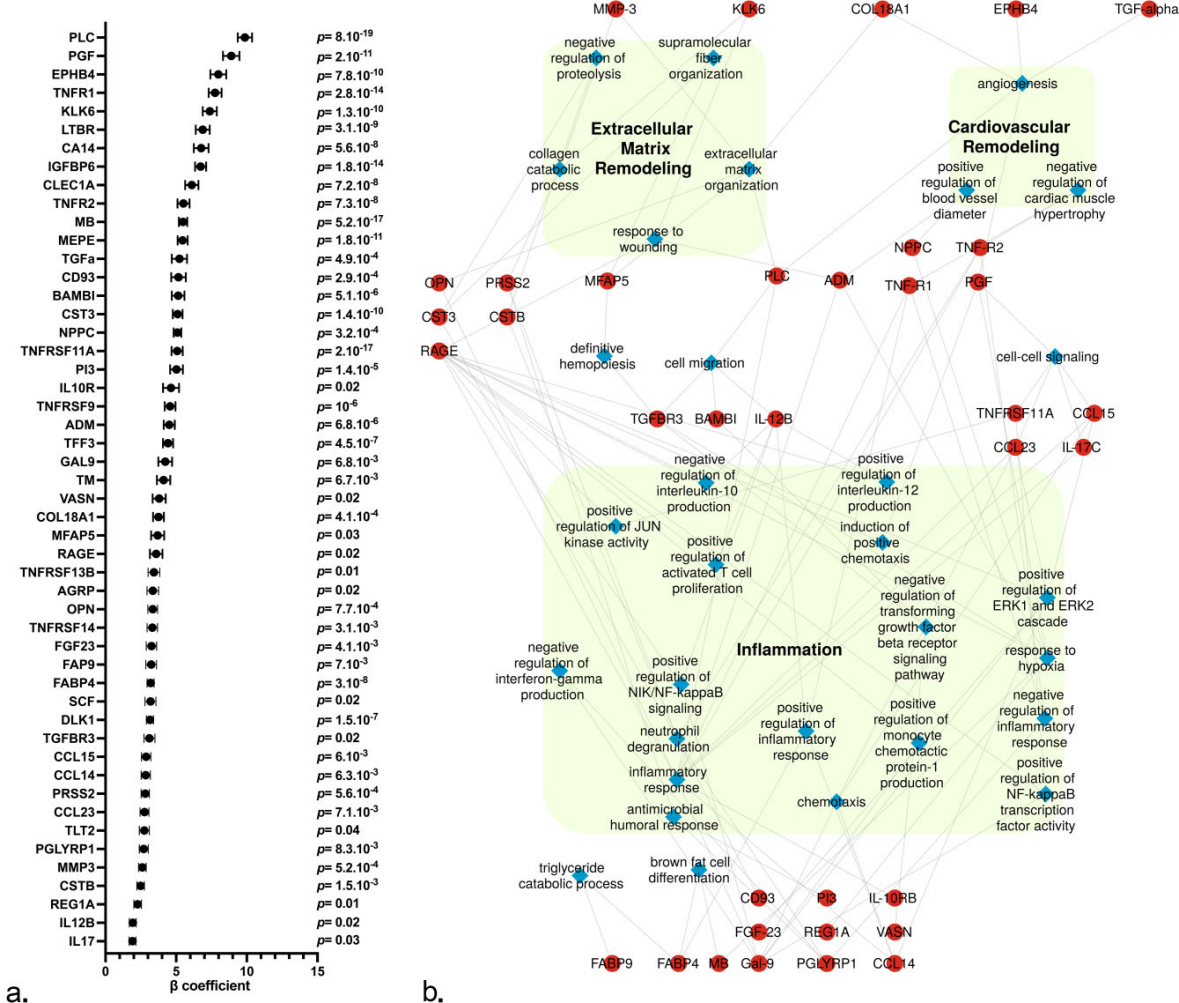
Associations between proteins levels measured at V4 and eGFR slope. (**a**) Forrest plot of proteins levels measured at V4 independently associated with eGFR slope. Multivariable linear regression model includes the following variables at V1: age, sex, BMI, current smoker, and eGFR. Dots represent β coefficient and standard error for each protein. *P* values (with Bonferroni correction) are indicated. (**b**) Network depiction of proteins co-expression modules that are positively correlated with eGFR slope at V4. Red dots represent proteins and blue diamonds represent biological process connecting the overrepresented proteins.

We analysed the proteomic signature associated with eGFR slope from V1 to V4 as a quantitative variable for each individual. The complete list of proteins measured both at V1 and V4 and their respective associations with eGFR slope are shown in the Supplemental Tables S2 and S3. After multivariable selection adjusted for the clinical features, the expression of six out of 445 proteins measured at V1 was found to be significantly and positively associated with eGFR slope and were therefore predictive of further kidney function decline: perlecan (PLC), matrix extracellular phosphoglycoprotein (MEPE), natriuretic peptide precursor C, tumour necrosis factor receptor superfamily member TNFRSF9, insulin-like growth factor binding protein-6, and cystatin C (Fig. 2). Moreover, after multivariable selection adjusted for the clinical features, the expression of 50 out of 445 proteins measured at V4 was found to be significantly and positively associated with eGFR slope, the top ones being PLC and placental growth factor (PGF) (Fig. 3). Conversely, none of the 119 765 genes expressions in the 887 individuals with available transcriptomic data at V4 was associated with eGFR slope (Supplemental Table S4).

**Figure 4:**
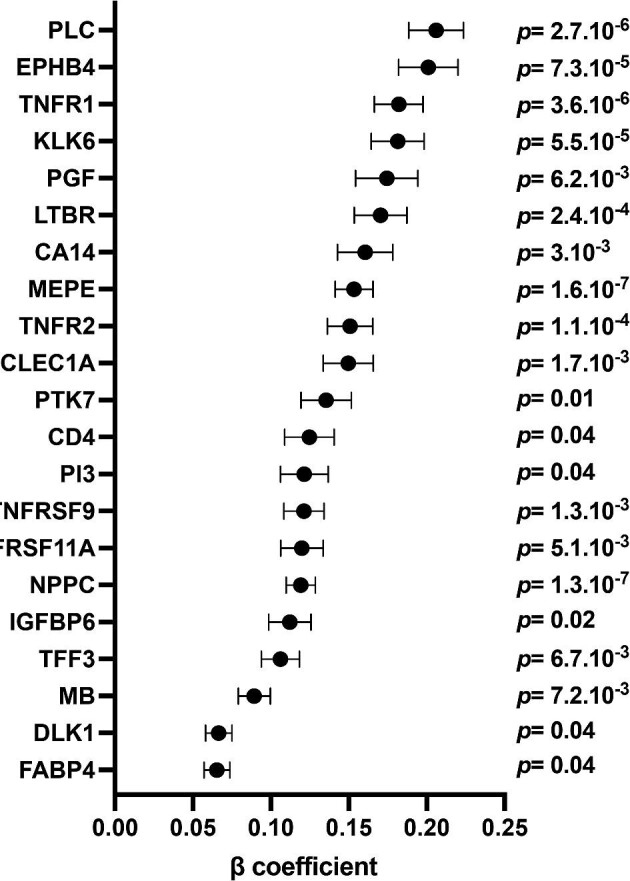
Associations between proteins levels measured at V4 and with ΔeGFR ≥ 15 ml/min/1.7 m^2^. Forrest plot of proteins levels measured at V4 independently associated with eGFR slope. Multivariable linear regression model includes the following variables at V1: age, sex, BMI, current smoker, and eGFR. Dots represent β coefficient and standard error for each protein. *P* values (with Bonferroni correction) are indicated.

**Figure 5:**
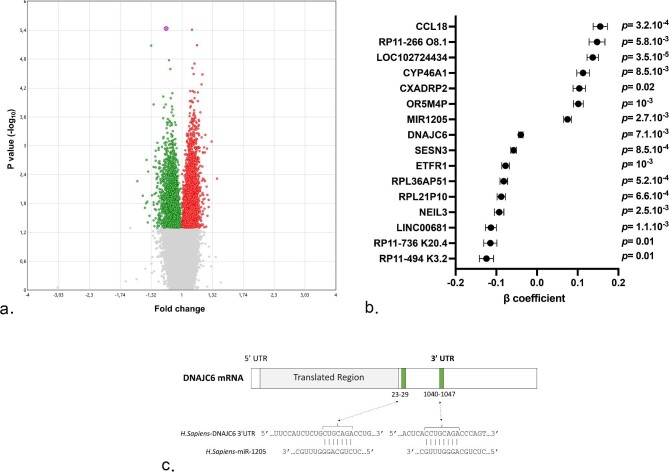
Associations between genes expressions at V4 and with ΔeGFR ≥ 15 ml/min/1.7 m^2^. (**a**) Volcano plot of genes differentially expressed at V4 when comparing groups of individuals with ΔeGFR < 15 ml/min/1.7 m^2^ (n = 718) and ΔeGFR ≥ 15 mL/min/1.7 m^2^ (*n* = 89). The *x* axis represents the mean fold change observed for each gene whereas the *y* axis displays the log_10_ of the *P* value. Each dot represents one gene. The green dots represent downregulated genes and red dots represent upregulated genes. (**b**) Forrest plots of genes measured at V4 independently associated with ΔeGFR ≥ 15 ml/min/1.7 m^2^. Multivariable logistic regression model includes the following variables at V1: age, sex, BMI, current smoker, and eGFR. Dots represent β coefficient and standard error for each gene. (**c**) Predicted duplex formation between miR-1205 and the 3′-UTR of the DNAJC6 mRNA by the miRDB and Targetscan algorithms.

The top overrepresented biological processes along with the network analysis results for the association between proteins measured at V1 and V4 and eGFR slope are summarized in Figs 4 and 5, and highlight three major features associated with new-onset kidney function decline: cardiovascular remodelling and extracellular matrix remodelling from V1, and inflammation at V4.

### Proteins/genes associated with ΔeGFR ≥ 15 ml/min/1.7 m^2^

To strengthen our analysis, we then compared the proteomic signature between groups of individuals with ΔeGFR ≥ 15 ml/min/1.7 m^2^ (*n* = 146) and ΔeGFR < 15 ml/min/1.7 m^2^ (*n* = 941) between V1 and V4. The complete list of proteins measured both at V1 and V4 and their respective associations with ΔeGFR ≥ 15 ml/min/1.7 m^2^ are shown in the Supplemental Tables S5 and S6. After multivariable selection adjusted for the clinical features, none of the proteins measured at V1 was independently associated with ΔeGFR ≥ 15 ml/min/1.7 m^2^. Conversely, the expression of 21 out of 445 proteins measured at V4 was found to be significantly and positively associated with new-onset kidney function decline (Fig. 4), the top ones including PLC, five members of the tumour necrosis factor receptor superfamily (TNFR1, TNFR2, TNFRSF9, TNFRSF11A, and LTBR), kallikrein 6 (KLK6), PGF, and MEPE. Areas under the ROC curves to identify ΔeGFR ≥ 15 ml/min/1.7 m^2^ at V4 were ≥75% for PLC, three members of the tumour necrosis factor receptor superfamily (TNFR1, TNFR2, and LTBR), ephrin type-B receptor 4 (EPHB4), KLK6, MEPE, and natriuretic peptide precursor C (Supplemental Fig. S2).

We then analysed the differential genes expression between groups of individuals with ΔeGFR ≥ 15 ml/min/1.7 m^2^ (*n* = 89) and ΔeGFR < 15 ml/min/1.7 m^2^ (*n* = 718) in the 887 individuals with available transcriptomic data at V4. The volcano plot is depicted in Fig. 5a and shows a slight, yet significant, association between new-onset kidney function decline and the genes expression profile. The expression of 16 out of the 119 765 genes was found to be independently associated with new-onset kidney function decline, including six protein-coding genes (Supplemental Table S7). Among the last, two were upregulated (CCL18 and CYP46A1), and four were downregulated (DNAJC6, SESN3, ETFR1, and NEIL3) in subjects with ΔeGFR ≥ 15 ml/min/1.7 m^2^ (Fig. 5b). Areas under the ROC curves to identify ΔeGFR ≥ 15 ml/min/1.7 m^2^ at V4 were ≥75% for all the genes herein identified (Supplemental Fig. S3).

Of particular interest, we noticed that the non-coding MIR1205, which expression is increased at V4 in subjects with new-onset kidney function decline, is predicted to be a potential negative regulator of DNAJC6 expression, which was found to be downregulated in this population. This suggests the existence of a new miRNA–mRNA pair associated with ΔeGFR ≥ 15 ml/min/1.7 m^2^ (Fig. 5c).

## DISCUSSION

The use of a multiomic approach represents a powerful tool to gain an in-depth understanding of the molecular events that modulate several diseases in an unbiased and exhaustive way [28–32]. We took advantage of the STANISLAS cohort to apply this methodology to the setting of new-onset GFR decrease in a unique, longitudinal, large, healthy population without comorbidities at baseline [8].

Our proteomic analysis unravelled 21 and 50 proteins measured at V4 that were independently and positively associated with ΔeGFR ≥ 15 ml/min/1.7 m^2^ and eGFR slope, respectively. Twenty proteins figured in the two lists, the protein having the highest β coefficient being PLC. PLC is a large proteoglycan involved in angiogenesis and extracellular matrix stabilization [33]. Interestingly, PLC plasma level was recently suggested as a surrogate of endothelial damage in CKD patients [34]. We also found PGF, a proatherogenic cytokine and biomarker for cardiovascular events, to be independently associated with eGFR slope, having the second highest β coefficient, while PGF level was previously reported to be increased in patients with decreased kidney function [35]. Taken together, the network analysis supported by our data at V4 highlights both cardiovascular and extracellular matrix remodelling and inflammation as key features of GFR decline.

Interestingly, some of those patterns seem to precede GFR decline. Indeed, six proteins measured at V1 were found to be associated with eGFR slope, the top one being PLC again. Our network analysis at V1 supports the idea of a cardiovascular and extracellular matrix remodelling that precedes GFR decline and strengthens the idea of a cross-link between kidney disease and accelerated vascular ageing. These findings bring more questions about the *primum movens* between the two phenomena [36].

The data from Dubin *et al.*, who recently described the proteomic patterns associated with GFR decline over 10 years in a cohort of patients with moderate CKD, highlighted the role of ephrin signalling in this setting [10]. Others have underscored the particular role of Ephrin-A4 [37, 38]. Interestingly, we found EPHB4, a receptor for ephrin involved in cardiovascular integrity [39], to be independently associated with GFR decline. In addition, Grams *et al.* reported associations of proteins levels with incidence of end-stage kidney disease in an older population and identified two members of the tumour necrosis factor receptor superfamily to correlate with renal function impairment [11], while circulating levels of these proteins have been previously reported to increase during later stages of CKD [40]. We report several members of this pro-inflammatory family of proteins to correlate with new-onset kidney function decline.

The familial structure our cohort also allowed to study the heritability for new-onset kidney function decline, which was found to be moderate. This suggests that small effects of many gene polymorphisms are involved in this process and that environmental factors are the most potent contributors to this variable.

Several gene expressions were independently associated with ΔeGFR ≥ 15 ml/min/1.7 m^2^ in our study. We report for the first time the positive association between CCL18 gene expression, one of the most highly expressed chemokines in human chronic inflammatory diseases and a biomarker of disease progression, and eGFR decline [41]. Additionally, we report for the first time a negative correlation between SESN3 gene expression, a stress-inducible protein known to protect against oxidative stress and hypoxia, and early kidney function impairment [42]. Similarly, reduced expression of the excision repair enzyme NEIL3, which was previously established as a driver of autoimmunity predisposition, was also associated with loss of renal function [43].

Of interest, both miRDB and Targetscan (two distinct algorithms used to predict miRNA targets) identified two binding sites for MIR1205 on the 3′-UTR region of the *DNAJC6* gene. DNAJC6 belongs to the evolutionarily conserved HSP40 proteins family of proteins and was recently reported to modulate energy metabolism [44]. Taken together with the fact that ΔeGFR ≥ 15 ml/min/1.7 m^2^ is associated with MIR1205 upregulation and DNAJC6 downregulation, these findings suggest the existence of a new miRNA–mRNA pair associated with new-onset GFR decline. Its pathophysiological significance remains to be established by future investigations.

The importance of early identification and intervention for CKD patients has been previously underscored [5]. However, besides limited accuracy, the available biomarkers (such as GFR and albuminuria) represent the consequence of the disease rather than the causal molecular pathophysiology, which further encouraged us to test the ability of the newly uncovered proteins/genes to identify new-onset GFR decline [45]. Interestingly, some of the herein highlighted proteins/genes reached an area over the ROC curve >0.75 to identify ΔeGFR ≥ 15 ml/min/1.7 m^2^ [46]. Whether some of them might represent future biomarkers or potential therapeutic targets to help diagnose kidney disease or slow down its progression remains to be established.

We acknowledge some limitations. First, this was an observational study, hence no causality can be inferred from our results. However, our findings should be regarded as hypothesis generating. Second, we used the eGFR formula to define new-onset kidney function decline and not direct GFR measurements. Nevertheless, we used the CKD-EPI equation, which has been demonstrated to perform more accurately especially at higher ranges of GFR, showing a 70% improvement at GFR > 60 ml/min/1.7 m^2^ compared to previous equations [16, 47]. Also, due to the 20-year follow-up design of our study, plasma creatinine levels were measured using different methods at V1 and V4, though the results from Jaffe and enzymatic methods have now been reported not to significantly differ [48, 49]. The relatively low GFR decline over 20 years in our cohort compared to what was previously reported in the general population may be explained by the selected healthy subjects included in our study [50]. Third, all circulating proteins were measured with the Olink^®^ technology, therefore no direct conversion to standard values is possible. Also, transcriptomic data were not available for all participants at V4 which could induce a selection bias, although baseline characteristics were similar in participants and nonparticipants at V4 [13]. Finally, it would be interesting to test if our transcriptomic findings could be confirmed at the protein scale, which was not allowed by our approach based on Olink^®^ panels measurements.

Our study provides for the first time a detailed and unbiased proteomic and transcriptomic characterization of new-onset eGFR decline over 20 years of follow-up in an initially healthy population. Our results, which highlight extracellular matrix and cardiovascular remodelling as well as inflammation as major features of early loss of kidney function, lay the foundation to further assess whether the proteins and genes herein identified may represent future biomarkers or potential therapeutic targets of interest to prevent for early CKD.

## Supplementary Material

sfae224_Supplemental_File

## Data Availability

The data collected for the study, including individual patient data and a data dictionary that define each field in the data set, will be made available as deidentified participant data to researchers who propose to use the data for individual patient data meta-analysis. Data will be shared following approval of the proposal by the corresponding author and a signed data access agreement.

## References

[1] Romagnani P, Remuzzi G, Glassock R et al. Chronic kidney disease. Nature Rev Disease Primers 2017;3:17088. 10.1038/nrdp.2017.88 29168475

[2] Foreman KJ, Marquez N, Dolgert A et al. Forecasting life expectancy, years of life lost, and all-cause and cause-specific mortality for 250 causes of death: reference and alternative scenarios for 2016–40 for 195 countries and territories. Lancet North Am Ed 2018;392:2052–90. 10.1016/S0140-6736(18)31694-5 PMC6227505 30340847

[3] Chronic Kidney Disease Prognosis Consortium, Matsushita K, van der Velde M et al. Association of estimated glomerular filtration rate and albuminuria with all-cause and cardiovascular mortality in general population cohorts: a collaborative meta-analysis. Lancet 2010;375:2073–81. 10.1016/S0140-6736(10)60674-5 PMC3993088 20483451

[4] Tonelli M, Wiebe N, Culleton B et al. Chronic kidney disease and mortality risk: a systematic review. J Am Soc Nephrol 2006;17:2034–47. 10.1681/ASN.2005101085 16738019

[5] Shlipak MG, Tummalapalli SL, Boulware LE et al. The case for early identification and intervention of chronic kidney disease: conclusions from a Kidney Disease: Improving Global Outcomes (KDIGO) controversies conference. Kidney Int 2021;99:34–47. 10.1016/j.kint.2020.10.012 33127436

[6] Hasin Y, Seldin M, Lusis A. Multi-omics approaches to disease. Genome Biol 2017;18:83. 10.1186/s13059-017-1215-1 28476144 PMC5418815

[7] Sammut S-J, Crispin-Ortuzar M, Chin S-F et al. Multi-omic machine learning predictor of breast cancer therapy response. Nature 2022;601:623–9. 10.1038/s41586-021-04278-5 34875674 PMC8791834

[8] Ferreira JP, Lamiral Z, Xhaard C et al. Circulating plasma proteins and new-onset diabetes in a population-based study: proteomic and genomic insights from the STANISLAS cohort. Eur J Endocrinol 2020;183:285–95. 10.1530/EJE-20-0246 32567559

[9] Joshi A, Rienks M, Theofilatos K et al. Systems biology in cardiovascular disease: a multiomics approach. Nature Rev Cardiol 2021;18:313–30. 10.1038/s41569-020-00477-1 33340009

[10] Dubin RF, Deo R, Ren Y et al. Proteomics of CKD progression in the chronic renal insufficiency cohort. Nat Commun 2023;14:6340. 10.1038/s41467-023-41642-7 37816758 PMC10564759

[11] Grams ME, Surapaneni A, Chen J et al. Proteins associated with risk of kidney function decline in the general population. J Am Soc Nephrol 2021;32:2291–302. 10.1681/ASN.2020111607 34465608 PMC8729856

[12] Ferreira JP, Girerd N, Bozec E et al. Cohort profile: rationale and design of the fourth visit of the STANISLAS cohort: a familial longitudinal population-based cohort from the Nancy region of France. Int J Epidemiol 2018;47:395–395j. 10.1093/ije/dyx240 29220499

[13] Xhaard C, de Villemereuil P, Benetos A et al. Shared heritability of blood pressure and pulse wave velocity: insights from the STANISLAS cohort. Hypertension 2023;80:1526–33. 10.1161/HYPERTENSIONAHA.122.20740 37165854

[14] Lameire NH, Levin A, Kellum JA et al. Harmonizing acute and chronic kidney disease definition and classification: report of a Kidney Disease: Improving Global Outcomes (KDIGO) Consensus Conference. Kidney Int 2021;100:516–26. 10.1016/j.kint.2021.06.028 34252450

[15] Inker LA, Collier W, Greene T et al. A meta-analysis of GFR slope as a surrogate endpoint for kidney failure. Nat Med 2023;29:1867–76. 10.1038/s41591-023-02418-0 37330614 PMC13037386

[16] Levey AS, Stevens LA, Schmid CH et al. A new equation to estimate glomerular filtration rate. Ann Intern Med 2009;150:604–12. 10.7326/0003-4819-150-9-200905050-00006 19414839 PMC2763564

[17] Lundberg M, Eriksson A, Tran B et al. Homogeneous antibody-based proximity extension assays provide sensitive and specific detection of low-abundant proteins in human blood. Nucleic Acids Res 2011;39:e102. 10.1093/nar/gkr424 21646338 PMC3159481

[18] Huttin O, Xhaard C, Dandine-Roulland C et al. Layer myocardial strain is the most heritable echocardiographic trait. European Heart J Cardiovasc Imag 2023;24:1394–1403. 10.1093/ehjci/jead146 37352124

[19] Thuillier Q, Behm-Ansmant I. Microarray analysis of whole-transcriptome RNAs including non-coding RNAs. Methods Mol Biol 2021;2300:143–64. 10.1007/978-1-0716-1386-3_14 33792879

[20] Ritchie ME, Phipson B, Wu D et al. limma powers differential expression analyses for RNA-sequencing and microarray studies. Nucleic Acids Res 2015;43:e47–. 10.1093/nar/gkv007 25605792 PMC4402510

[21] Xhaard C, Dandine-Roulland C, Villemereuil Pd et al. Heritability of a resting heart rate in a 20-year follow-up family cohort with GWAS data: insights from the STANISLAS cohort. Eur J Prev Cardiol 2021;28:1334–41. 10.1177/2047487319890763 34647585

[22] Lopez-Sublet M, Merkling T, Girerd N et al. Birth weight and subclinical cardiovascular and renal damage in a population-based study (the STANISLAS cohort study). J Hypertens 2023;41:1040–50. 10.1097/HJH.0000000000003438 37071444

[23] Gorski M, Rasheed H, Teumer A et al. Genetic loci and prioritization of genes for kidney function decline derived from a meta-analysis of 62 longitudinal genome-wide association studies. Kidney Int 2022;102:624–39. 10.1016/j.kint.2022.05.021 35716955 PMC10034922

[24] Storey JD, Tibshirani R. Statistical significance for genomewide studies. Proc Nat Acad Sci USA 2003;100:9440–5. 10.1073/pnas.1530509100 12883005 PMC170937

[25] Girerd N, Bresso E, Devignes M-D et al. Insulin-like growth factor binding protein 2: a prognostic biomarker for heart failure hardly redundant with natriuretic peptides. Int J Cardiol 2020;300:252–4. 10.1016/j.ijcard.2019.11.100 31761405

[26] UniProt Consortium T . UniProt: the universal protein knowledgebase. Nucleic Acids Res 2018;46:2699. 10.1093/nar/gky092 29425356 PMC5861450

[27] Gillespie M, Jassal B, Stephan R et al. The reactome pathway knowledgebase 2022. Nucleic Acids Res 2022;50:D687–D92. 10.1093/nar/gkab1028 34788843 PMC8689983

[28] Shannon P, Markiel A, Ozier O et al. Cytoscape: a software environment for integrated models of biomolecular interaction networks. Genome Res 2003;13:2498–504. 10.1101/gr.1239303 14597658 PMC403769

[29] Arnett DK, Claas SA. Omics of blood pressure and hypertension. Circ Res 2018;122:1409–19. 10.1161/CIRCRESAHA.118.311342 29748366

[30] Lu M, Zhan X. The crucial role of multiomic approach in cancer research and clinically relevant outcomes. EPMA J 2018;9:77–102. 10.1007/s13167-018-0128-8 29515689 PMC5833337

[31] Marttinen M, Paananen J, Neme A et al. A multiomic approach to characterize the temporal sequence in Alzheimer's disease-related pathology. Neurobiol Dis 2019;124:454–68. 10.1016/j.nbd.2018.12.009 30557660

[32] Tayanloo-Beik A, Roudsari PP, Rezaei-Tavirani M et al. Diabetes and heart failure: multi-omics approaches. Front Physiol 2021;12:705424. 10.3389/fphys.2021.705424 34421642 PMC8378451

[33] Hayes AJ, Farrugia BL, Biose IJ et al., A multi-functional, cell-instructive, matrix-stabilizing proteoglycan with roles in tissue development has relevance to connective tissue repair and regeneration. Front Cell Dev Biol 2022;10:856261. 10.3389/fcell.2022.856261 35433700 PMC9010944

[34] Valera G, Figuer A, Caro J et al. Plasma glycocalyx pattern: a mirror of endothelial damage in chronic kidney disease. Clin Kidney J 2023;16:1278–87. 10.1093/ckj/sfad051 37529650 PMC10387401

[35] Zakiyanov O, Kalousová M, Zima T et al. Placental growth factor in patients with decreased renal function. Ren Fail 2011;33:291–7. 10.3109/0886022X.2011.560402 21401353

[36] Hobson S, Arefin S, Witasp A et al. Accelerated vascular aging in CKD: the potential for novel therapies. Circ Res 2023;132:950–69. 10.1161/CIRCRESAHA.122.321751 37053277

[37] Jalal D, Sanford B, Renner B et al. Detection of pro angiogenic and inflammatory biomarkers in patients with CKD. Sci Rep 2021;11:8786. 10.1038/s41598-021-87710-0 33888746 PMC8062467

[38] Zabetian A, Coca SG. Plasma and urine biomarkers in chronic kidney disease: closer to clinical application. Curr Opin Nephrol Hypertens 2021;30:531–7. 10.1097/MNH.0000000000000735 34475336 PMC8490303

[39] Luxán G, Stewen J, Díaz N et al. Endothelial EphB4 maintains vascular integrity and transport function in adult heart. eLife 2019;8:e45863. 10.7554/eLife.45863 31782728 PMC6884395

[40] Gohda T, Niewczas MA, Ficociello LH et al. Circulating TNF receptors 1 and 2 predict stage 3 CKD in type 1 diabetes. J Am Soc Nephrol 2012;23:516–24. 10.1681/ASN.2011060628 22266664 PMC3294299

[41] Cantero-Navarro E, Rayego-Mateos S, Orejudo M et al. Role of macrophages and related cytokines in kidney disease. Front Med 2021;8:688060. 10.3389/fmed.2021.688060 PMC8295566 34307414

[42] Chen Y, Huang T, Yu Z et al. The functions and roles of sestrins in regulating human diseases. Cell Mol Biol Lett 2022;27:2. 34979914 10.1186/s11658-021-00302-8 PMC8721191

[43] Massaad MJ, Zhou J, Tsuchimoto D et al. Deficiency of base excision repair enzyme NEIL3 drives increased predisposition to autoimmunity. J Clin Invest 2016;126:4219–36. 10.1172/JCI85647 27760045 PMC5096910

[44] Kim J, Lee M. RMR-related DNAJC6 expression suppresses adipogenesis in 3T3-L1 cells. Cells 2022;11:1331. 10.3390/cells11081331 35456010 PMC9031806

[45] Siwy J, Mischak H, Beige J et al. Biomarkers for early detection of kidney disease: a call for pathophysiological relevance. Kidney Int 2021;99:1240–1. 10.1016/j.kint.2021.02.008 33892861

[46] Ray P, Le Manach Y, Riou B et al. Statistical evaluation of a biomarker. Anesthesiology 2010;112:1023–40. 10.1097/ALN.0b013e3181d47604 20234303

[47] Stevens LA, Schmid CH, Greene T et al. Comparative performance of the CKD Epidemiology Collaboration (CKD-EPI) and the Modification of Diet in Renal Disease (MDRD) Study equations for estimating GFR levels above 60 mL/min/1.73 m^2^. Am J Kidney Dis 2010;56:486–95. 10.1053/j.ajkd.2010.03.026 20557989 PMC2926290

[48] Osmic-Husni A, Hukic F, Saric MP. Comparison of Jaffe method and enzymatic method at measuring serum creatinine level, creatinine clearance and estimated glomerular filtration rate. Materia Socio-Medica 2023;35:108–12. 10.5455/msm.2023.35.113-117 PMC10495157 37701344

[49] Küme T, Sağlam B, Ergon C et al. Evaluation and comparison of Abbott Jaffe and enzymatic creatinine methods: could the old method meet the new requirements? J Clin Lab Anal 2018;32:e22168. 10.1002/jcla.22168 28205269 PMC6816857

[50] Waas T, Schulz A, Lotz J et al. Distribution of estimated glomerular filtration rate and determinants of its age dependent loss in a German population-based study. Sci Rep 2021;11:10165. 10.1038/s41598-021-89442-7 33986324 PMC8119940

