# Supplementary material for: Multiomic profiling of new-onset kidney function decline: insights from the STANISLAS study cohort with a 20-year follow-up
Source: Clin Kidney J. 2024 Jul 18;17(8):sfae224. doi: 10.1093/ckj/sfae224 (PMC11317839; doi:10.1093/ckj/sfae224)
Supplement: sfae224_Supplemental_File [file sfae224_supplemental_file.pdf]

Multiomic Profiling of Early Kidney Function Decline:

Insights from the STANISLAS Study Cohort with a 20-Year Follow-up

Supplemental Material

Supplemental Table 1. Cardiovascular features at V4 in the study population and according to eGFR decline status. (P2)

Supplemental Table 2: Associations between proteins levels measured at V1 and eGFR slope. (P24)

Supplemental Table 3: Associations between proteins levels measured at V4 and eGFR slope. (P34)

Supplemental Table 4: Associations between genes expressions measured at V4 and eGFR slope. (P44)

Supplemental Table 5: Associations between proteins levels measured at V1 and ΔeGFR≥15mL/min/1.7m^2. (P3)

Supplemental Table 6: Associations between proteins levels measured at V4 and ΔeGFR≥15mL/min/1.7m^2. (P13)

Supplemental Table 7: Associations between genes expressions measured at V4 and ΔeGFR≥15mL/min/1.7m^2. (P23)

Supplemental Figure 1: Kidney function decline over 20 years. (P45)

Supplemental Figure 2: Predictive ability of proteins levels measured at V4 for new-onset kidney function decline. (P46)

Supplemental Figure 3: Predictive ability of genes expressions measured at V4 for new-onset kidney function decline. (P47)

Supplemental Table 1. Cardiovascular features at V4 in the study population and according to eGFR decline status

	Overall (n=1087)	ΔeGFR<15mL/min/1.7m^2 (n=941)	ΔeGFR≥15mL/min/1.7m^2 (n=146)	P value
Pulse Wave Velocity (m/s)	9.0±1.8	9.0±1.9	9.0±1.5	0.68
Carotid intima media thickness (μm)	686.2±139.8	687.4±139.6	678.3±141.3	0.47
Index left ventricular mass (g/m²)	74.4±20.1	79.2±19.6	80.7±23.1	0.46
Left ventricular hypertrophy	186(17.1)	163(17.3)	23(15.8)	0.73
e' septal (cm/s)	8.7±2.3	8.7±2.4	8.8±2.2	0.71
e' lateral (cm/s)	11.1±3.1	11.1±3.1	11.0±3.0	0.76
Left atrial volume index (mL)	23.3±7.9	23.4±8.0	23.1±7.4	0.68
e/a	1.03±0.32	1.02±0.32	1.07±0.33	0.09
Deceleration Time	218±55	217±53	222±64	0.36
e/e'	6.95±1.91	6.94±1.88	6.99±2.15	0.81

Variables are expressed as number(percentage) or mean±standard deviation. Left ventricular hypertrophy is defined by an indexed left ventricular mass≥ 89 or 103 g/m² for women and men, respectively. Multivariable logistic regression model includes the following variables at V1: age, sex, body mass index (BMI), current smoker and eGFR. eGFR, estimated glomerular filtration rate.

Supplemental Table 2: Associations between proteins levels measured at V1 and eGFR slope.

Multivariable linear regression models included including the following variables at V1: age, sex, body mass index, current smoker and eGFR.

Protein at V1	β coefficient	Standard error	P value	Bonferroni P value
PLC	4,83	1,14	2,67E-05	1,19E-02
IGFBP6	3,57	0,86	3,44E-05	1,54E-02
NPPC	2,47	0,60	3,70E-05	1,65E-02
TNFRSF9	3,56	0,86	4,01E-05	1,79E-02
CST3	3,03	0,76	6,79E-05	3,04E-02
MEPE	3,04	0,77	8,13E-05	3,63E-02
GDNF	2,82	0,77	2,51E-04	1,12E-01
TNFR1	3,93	1,08	2,73E-04	1,22E-01
CD93	3,78	1,09	5,64E-04	2,52E-01
CD6	2,41	0,72	8,73E-04	3,90E-01
PGF	4,20	1,28	1,09E-03	4,86E-01
SCF	2,50	0,77	1,25E-03	5,57E-01
TNFR2	3,06	0,97	1,56E-03	6,99E-01
TGFalpha	3,12	0,99	1,59E-03	7,11E-01
PAPPA	1,72	0,55	1,71E-03	7,63E-01
COL1A1	2,66	0,85	1,75E-03	7,84E-01
IL10RB	3,57	1,14	1,84E-03	8,22E-01
PDL1	1,96	0,65	2,52E-03	1,00E+00
VASN	2,96	0,98	2,58E-03	1,00E+00
CD5	3,15	1,04	2,58E-03	1,00E+00
CA12	2,89	0,97	3,09E-03	1,00E+00
CSF1	3,96	1,34	3,11E-03	1,00E+00
LHB	0,91	0,31	3,27E-03	1,00E+00
LTBR	3,16	1,09	3,82E-03	1,00E+00
VCAM1	2,45	0,85	4,14E-03	1,00E+00
CCL14	2,03	0,71	4,51E-03	1,00E+00
CA14	3,19	1,16	6,20E-03	1,00E+00
DLK1	1,44	0,54	7,48E-03	1,00E+00
OPN	1,99	0,74	7,56E-03	1,00E+00
EPHB4	3,19	1,20	7,78E-03	1,00E+00
IL10	1,60	0,61	8,44E-03	1,00E+00
ICAM1	2,19	0,83	8,55E-03	1,00E+00
CCL23	1,80	0,68	8,66E-03	1,00E+00
hOSCAR	3,33	1,27	9,14E-03	1,00E+00
CALCA	1,21	0,47	1,03E-02	1,00E+00
PLTP	2,62	1,02	1,08E-02	1,00E+00
IL18BP	2,55	1,01	1,13E-02	1,00E+00

TNFB	1,77	0,71	1,28E-02	1,00E+00
PGLYRP1	1,89	0,77	1,36E-02	1,00E+00
MMP2	2,48	1,00	1,36E-02	1,00E+00
BID	-1,88	0,76	1,38E-02	1,00E+00
FABP9	1,83	0,74	1,40E-02	1,00E+00
AXL	2,48	1,02	1,51E-02	1,00E+00
SPARCL1	2,34	0,96	1,52E-02	1,00E+00
TIMP1	2,05	0,85	1,57E-02	1,00E+00
BOC	2,78	1,16	1,70E-02	1,00E+00
NCF2	1,35	0,57	1,86E-02	1,00E+00
NOTCH1	2,57	1,09	1,92E-02	1,00E+00
ST2	1,33	0,57	1,94E-02	1,00E+00
FCGR2A	1,23	0,53	1,96E-02	1,00E+00
ENG	2,46	1,06	2,03E-02	1,00E+00
MPO	1,79	0,77	2,04E-02	1,00E+00
WAS	-0,76	0,33	2,22E-02	1,00E+00
THBS4	1,35	0,60	2,33E-02	1,00E+00
PRSS27	1,64	0,73	2,37E-02	1,00E+00
DCN	3,42	1,52	2,42E-02	1,00E+00
CLEC1A	2,31	1,02	2,46E-02	1,00E+00
TM	2,58	1,15	2,49E-02	1,00E+00
PCOLCE	1,65	0,74	2,54E-02	1,00E+00
BetaNGF	2,53	1,15	2,81E-02	1,00E+00
PDL2	2,06	0,94	2,82E-02	1,00E+00
PSPD	0,96	0,44	2,85E-02	1,00E+00
TLT2	1,81	0,83	3,03E-02	1,00E+00
IL18R1	1,75	0,81	3,08E-02	1,00E+00
ALCAM	2,97	1,39	3,22E-02	1,00E+00
IL12B	1,15	0,54	3,23E-02	1,00E+00
TRAIL	2,19	1,02	3,28E-02	1,00E+00
IGLC2	1,26	0,59	3,32E-02	1,00E+00
LILRB5	0,80	0,38	3,38E-02	1,00E+00
MMP3	1,20	0,57	3,40E-02	1,00E+00
CDH5	2,13	1,00	3,41E-02	1,00E+00
COL18A1	1,71	0,83	3,91E-02	1,00E+00
ITGB1BP1	-2,70	1,31	3,92E-02	1,00E+00
TGFBR3	1,65	0,80	3,93E-02	1,00E+00
MB	1,31	0,64	4,10E-02	1,00E+00
TNFRSF13B	1,91	0,94	4,29E-02	1,00E+00
IL16	1,56	0,77	4,34E-02	1,00E+00
MMP10	0,96	0,48	4,41E-02	1,00E+00
MAX	-0,76	0,38	4,74E-02	1,00E+00
IL1ra	1,18	0,59	4,79E-02	1,00E+00
CR2	1,16	0,59	4,85E-02	1,00E+00
MFAP5	1,83	0,93	4,92E-02	1,00E+00
IL4RA	2,14	1,10	5,25E-02	1,00E+00

RAGE	1,83	0,96	5,64E-02	1,00E+00
CA1	0,93	0,50	5,96E-02	1,00E+00
TRAP	-1,61	0,86	6,22E-02	1,00E+00
PDCD1	1,40	0,75	6,27E-02	1,00E+00
CSTB	1,22	0,66	6,35E-02	1,00E+00
CD8A	0,92	0,50	6,52E-02	1,00E+00
NRP1	3,38	1,84	6,60E-02	1,00E+00
LILRB1	1,69	0,92	6,67E-02	1,00E+00
ICAM2	1,50	0,82	6,71E-02	1,00E+00
UPAR	1,73	0,95	6,88E-02	1,00E+00
TNFRSF10C	1,05	0,58	7,00E-02	1,00E+00
EFEMP1	1,43	0,80	7,44E-02	1,00E+00
PRTN3	1,17	0,66	7,50E-02	1,00E+00
THBS2	2,84	1,59	7,50E-02	1,00E+00
TRANCE	0,87	0,49	7,74E-02	1,00E+00
CTSD	-1,54	0,87	7,84E-02	1,00E+00
CPA1	-0,83	0,47	7,88E-02	1,00E+00
TNFRSF11A	1,72	0,98	7,96E-02	1,00E+00
KLK6	1,71	0,98	8,03E-02	1,00E+00
QPCT	2,41	1,40	8,53E-02	1,00E+00
Notch3	1,51	0,88	8,70E-02	1,00E+00
NCAM1	1,41	0,83	9,14E-02	1,00E+00
RETN	1,22	0,73	9,65E-02	1,00E+00
SELL	1,35	0,81	9,73E-02	1,00E+00
LIF	-1,21	0,73	1,03E-01	1,00E+00
SHPS1	1,32	0,81	1,03E-01	1,00E+00
IL20	-1,70	1,04	1,05E-01	1,00E+00
Gal9	1,81	1,14	1,11E-01	1,00E+00
TIE1	2,15	1,35	1,12E-01	1,00E+00
CA4	1,67	1,05	1,14E-01	1,00E+00
AOC3	1,35	0,86	1,15E-01	1,00E+00
CD59	2,63	1,66	1,15E-01	1,00E+00
MAEA	-1,06	0,68	1,20E-01	1,00E+00
CRH	0,57	0,37	1,21E-01	1,00E+00
IL2RA	1,14	0,75	1,28E-01	1,00E+00
MET	1,63	1,07	1,31E-01	1,00E+00
PON3	-0,83	0,55	1,31E-01	1,00E+00
TWEAK	1,58	1,05	1,32E-01	1,00E+00
MBL2	0,35	0,24	1,33E-01	1,00E+00
GDF15	0,99	0,66	1,33E-01	1,00E+00
ERBB2IP	-0,60	0,40	1,35E-01	1,00E+00
IL17D	1,71	1,15	1,38E-01	1,00E+00
IGFBP2	0,70	0,48	1,40E-01	1,00E+00
CASP3	-0,50	0,34	1,44E-01	1,00E+00
BAMBI	1,19	0,82	1,49E-01	1,00E+00
ICAM3	1,34	0,93	1,49E-01	1,00E+00

TNFRSF14	1,13	0,79	1,56E-01	1,00E+00
CXCL10	0,57	0,41	1,64E-01	1,00E+00
CLSPN	-3,13	2,24	1,65E-01	1,00E+00
LTA4H	15,97	9,84	1,65E-01	1,00E+00
LRP1	-1,12	0,81	1,68E-01	1,00E+00
DPP6	1,38	1,00	1,68E-01	1,00E+00
vWF	0,70	0,51	1,70E-01	1,00E+00
CD244	1,04	0,77	1,74E-01	1,00E+00
CCL19	0,52	0,38	1,75E-01	1,00E+00
IL17RA	0,84	0,62	1,76E-01	1,00E+00
OSMR	1,86	1,38	1,79E-01	1,00E+00
IL1RT1	1,60	1,19	1,79E-01	1,00E+00
IL24	-1,18	0,89	1,86E-01	1,00E+00
DEFA1	-6,69	4,37	1,86E-01	1,00E+00
OSM	0,51	0,39	1,90E-01	1,00E+00
CAPG	0,84	0,65	1,94E-01	1,00E+00
CAPG	0,84	0,65	1,94E-01	1,00E+00
CX3CL1	1,17	0,91	1,98E-01	1,00E+00
FS	-0,65	0,51	2,03E-01	1,00E+00
STX8	-0,58	0,45	2,06E-01	1,00E+00
CRTAC1	0,71	0,56	2,06E-01	1,00E+00
TSLP	1,54	1,17	2,08E-01	1,00E+00
IL13	0,86	0,68	2,10E-01	1,00E+00
AIFM1	-0,39	0,31	2,11E-01	1,00E+00
SMAD1	-0,44	0,36	2,13E-01	1,00E+00
TNF	0,79	0,63	2,14E-01	1,00E+00
GIF	0,54	0,43	2,15E-01	1,00E+00
LIFR	1,59	1,29	2,17E-01	1,00E+00
PIgR	3,12	2,56	2,23E-01	1,00E+00
DNER	-1,71	1,40	2,23E-01	1,00E+00
EDIL3	2,83	2,33	2,25E-01	1,00E+00
PAI	-0,60	0,49	2,26E-01	1,00E+00
CD46	0,63	0,52	2,27E-01	1,00E+00
PLXDC1	1,37	1,15	2,33E-01	1,00E+00
PTX3	0,81	0,68	2,36E-01	1,00E+00
ADGRG1	1,14	0,96	2,37E-01	1,00E+00
IL1RL2	0,75	0,64	2,39E-01	1,00E+00
TNC	0,77	0,66	2,42E-01	1,00E+00
IGFBP3	0,95	0,81	2,42E-01	1,00E+00
TGFBI	0,82	0,70	2,43E-01	1,00E+00
DSG4	0,71	0,60	2,43E-01	1,00E+00
LOX1	0,73	0,63	2,45E-01	1,00E+00
CHL1	0,97	0,84	2,47E-01	1,00E+00
FGF21	-0,28	0,24	2,51E-01	1,00E+00
NTproBNP	0,54	0,47	2,51E-01	1,00E+00
ANGPTL3	0,85	0,74	2,51E-01	1,00E+00

CTSZ	1,04	0,91	2,54E-01	1,00E+00
PDGFsubunitB	-0,62	0,54	2,54E-01	1,00E+00
ITGB1BP2	-0,34	0,30	2,59E-01	1,00E+00
CSNK1D	0,80	0,71	2,62E-01	1,00E+00
IL27	0,88	0,79	2,63E-01	1,00E+00
PXN	0,89	0,80	2,63E-01	1,00E+00
LPL	-0,55	0,49	2,64E-01	1,00E+00
CALR	-1,63	1,46	2,64E-01	1,00E+00
STXBP3	-0,59	0,52	2,64E-01	1,00E+00
GALNT10	-1,35	1,21	2,65E-01	1,00E+00
JAMA	-0,36	0,32	2,66E-01	1,00E+00
CTRC	0,54	0,49	2,67E-01	1,00E+00
AGR2	0,50	0,46	2,69E-01	1,00E+00
MMP7	-0,57	0,52	2,70E-01	1,00E+00
TNFSF13B	1,13	1,03	2,73E-01	1,00E+00
CEACAM8	0,63	0,57	2,75E-01	1,00E+00
TIE2	1,54	1,42	2,76E-01	1,00E+00
LILRB2	0,87	0,80	2,81E-01	1,00E+00
SELP	-0,44	0,41	2,81E-01	1,00E+00
IL7R	0,85	0,79	2,82E-01	1,00E+00
GP1BA	0,40	0,38	2,82E-01	1,00E+00
NID1	0,68	0,63	2,85E-01	1,00E+00
ENTPD6	-1,27	1,18	2,85E-01	1,00E+00
CXCL5	0,36	0,34	2,89E-01	1,00E+00
SAA4	0,57	0,54	2,93E-01	1,00E+00
ADM	0,96	0,91	2,93E-01	1,00E+00
BNP	-2,18	2,07	2,94E-01	1,00E+00
TNXB	1,40	1,34	2,97E-01	1,00E+00
PRKAB1	-0,42	0,40	2,99E-01	1,00E+00
TFPI	-0,98	0,95	3,00E-01	1,00E+00
VASH1	-0,40	0,39	3,03E-01	1,00E+00
LAT2	-0,31	0,30	3,04E-01	1,00E+00
FCGR3B	0,57	0,57	3,18E-01	1,00E+00
SERPINA9	0,59	0,59	3,18E-01	1,00E+00
CXCL9	0,40	0,41	3,20E-01	1,00E+00
PTPRS	2,07	2,09	3,21E-01	1,00E+00
IL8	-0,55	0,56	3,24E-01	1,00E+00
IL10RA	0,49	0,50	3,28E-01	1,00E+00
COMP	0,70	0,72	3,28E-01	1,00E+00
CCL25	-0,56	0,58	3,30E-01	1,00E+00
AGRP	0,79	0,82	3,36E-01	1,00E+00
PRELP	1,82	1,89	3,36E-01	1,00E+00
IL20RA	-0,90	0,95	3,45E-01	1,00E+00
PTK7	0,90	0,96	3,50E-01	1,00E+00
REG1A	0,53	0,57	3,50E-01	1,00E+00
ACE2	-0,58	0,62	3,53E-01	1,00E+00

PECAM1	-0,37	0,41	3,55E-01	1,00E+00
IL1alpha	1,31	1,41	3,59E-01	1,00E+00
PDGFsubunitA	-0,41	0,45	3,61E-01	1,00E+00
MAGED1	-0,90	0,99	3,62E-01	1,00E+00
IL7	-0,45	0,50	3,63E-01	1,00E+00
FKBP1B	-0,31	0,35	3,71E-01	1,00E+00
LYVE1	0,69	0,77	3,74E-01	1,00E+00
PCSK9	-0,79	0,89	3,78E-01	1,00E+00
KIT	0,67	0,76	3,79E-01	1,00E+00
SLAMF1	0,68	0,78	3,80E-01	1,00E+00
GH	0,14	0,16	3,84E-01	1,00E+00
NBN	0,56	0,65	3,89E-01	1,00E+00
ITGB2	0,79	0,93	3,91E-01	1,00E+00
TF	1,00	1,17	3,93E-01	1,00E+00
IL18	-0,55	0,65	3,94E-01	1,00E+00
HBEGF	-0,38	0,44	3,94E-01	1,00E+00
TNNI3	-0,46	0,54	3,95E-01	1,00E+00
IL6	0,37	0,44	3,95E-01	1,00E+00
ATP6AP2	1,10	1,30	3,97E-01	1,00E+00
RARRES1	-1,13	1,34	3,99E-01	1,00E+00
TNFSF14	0,45	0,54	4,02E-01	1,00E+00
PTPRJ	-0,32	0,39	4,04E-01	1,00E+00
IL1RT2	0,87	1,05	4,06E-01	1,00E+00
IGFBP1	0,29	0,35	4,07E-01	1,00E+00
VSIG2	0,64	0,77	4,08E-01	1,00E+00
GP6	-0,42	0,51	4,08E-01	1,00E+00
RASA1	0,65	0,79	4,14E-01	1,00E+00
CNDP1	-0,56	0,69	4,14E-01	1,00E+00
FAP	0,79	0,97	4,15E-01	1,00E+00
CES1	-0,48	0,60	4,23E-01	1,00E+00
FOSB	0,41	0,52	4,27E-01	1,00E+00
CXCL1	0,40	0,50	4,28E-01	1,00E+00
NEMO	-0,29	0,37	4,33E-01	1,00E+00
ST3GAL1	-0,44	0,56	4,33E-01	1,00E+00
PRKRA	-0,40	0,52	4,36E-01	1,00E+00
MAP4K5	-0,20	0,25	4,38E-01	1,00E+00
PON2	-0,48	0,63	4,47E-01	1,00E+00
TIMD4	0,47	0,61	4,48E-01	1,00E+00
FAS	0,71	0,94	4,49E-01	1,00E+00
PTN	-0,42	0,55	4,49E-01	1,00E+00
TGM2	0,45	0,59	4,50E-01	1,00E+00
BMP6	0,52	0,70	4,61E-01	1,00E+00
IgGFcreceptorIIb	-0,29	0,40	4,63E-01	1,00E+00
SERPINA12	0,20	0,27	4,71E-01	1,00E+00
CES2	-1,29	1,81	4,76E-01	1,00E+00
FGF5	1,35	1,89	4,77E-01	1,00E+00

EpCAM	-0,22	0,31	4,78E-01	1,00E+00
GDF2	-0,69	0,97	4,78E-01	1,00E+00
PRCP	-3,33	4,35	4,79E-01	1,00E+00
CCL15	0,46	0,65	4,80E-01	1,00E+00
PPM1B	-2,23	3,09	4,80E-01	1,00E+00
GT	-0,41	0,59	4,81E-01	1,00E+00
ADA	0,59	0,85	4,83E-01	1,00E+00
CNTN1	0,77	1,11	4,85E-01	1,00E+00
PSGL1	0,85	1,22	4,87E-01	1,00E+00
SIRT5	0,76	1,09	4,87E-01	1,00E+00
MMP9	0,32	0,47	4,90E-01	1,00E+00
NOS3	0,50	0,73	4,95E-01	1,00E+00
NUCB2	-0,28	0,42	5,00E-01	1,00E+00
TFF3	-0,20	0,30	5,01E-01	1,00E+00
GAS6	0,53	0,79	5,03E-01	1,00E+00
CHI3L1	0,32	0,49	5,05E-01	1,00E+00
uPA	-0,56	0,84	5,06E-01	1,00E+00
NUB1	-0,24	0,36	5,07E-01	1,00E+00
DPP4	0,56	0,85	5,07E-01	1,00E+00
FABP2	-0,28	0,43	5,10E-01	1,00E+00
LDLreceptor	-0,39	0,59	5,13E-01	1,00E+00
ENTPD2	-1,19	1,82	5,15E-01	1,00E+00
ENAH	0,42	0,67	5,24E-01	1,00E+00
ADAMTS13	-1,24	1,95	5,24E-01	1,00E+00
REG3A	10,79	16,11	5,28E-01	1,00E+00
FGR	0,30	0,47	5,29E-01	1,00E+00
IL22RA1	-2,14	3,14	5,33E-01	1,00E+00
IGFBP7	0,62	1,01	5,35E-01	1,00E+00
SERPINA7	0,44	0,71	5,36E-01	1,00E+00
OPG	-0,56	0,91	5,37E-01	1,00E+00
AZU1	0,29	0,48	5,38E-01	1,00E+00
CCL5	0,19	0,31	5,43E-01	1,00E+00
ARTN	-0,44	0,73	5,43E-01	1,00E+00
IL18	-0,39	0,65	5,45E-01	1,00E+00
SRC	0,71	1,18	5,48E-01	1,00E+00
PARP1	0,41	0,69	5,51E-01	1,00E+00
AMBP	1,05	1,76	5,51E-01	1,00E+00
SPON1	-1,07	1,80	5,54E-01	1,00E+00
CCL28	0,45	0,77	5,55E-01	1,00E+00
INPPL1	-0,19	0,32	5,56E-01	1,00E+00
IL17A	0,37	0,63	5,61E-01	1,00E+00
CTSL1	0,61	1,07	5,65E-01	1,00E+00
VEGFC	0,28	0,49	5,67E-01	1,00E+00
PDGFC	0,53	0,93	5,70E-01	1,00E+00
ANG	0,29	0,51	5,71E-01	1,00E+00
E4BP1	-0,21	0,37	5,74E-01	1,00E+00

S2	0,79	1,42	5,79E-01	1,00E+00
TRAILR2	0,39	0,70	5,81E-01	1,00E+00
S1	1,45	2,62	5,82E-01	1,00E+00
YES1	-0,15	0,28	5,84E-01	1,00E+00
HAOX1	-0,12	0,22	5,85E-01	1,00E+00
IFNgamma	6,22	11,12	5,89E-01	1,00E+00
C1QTNF1	0,33	0,62	5,92E-01	1,00E+00
CCL20	-0,18	0,35	5,98E-01	1,00E+00
CXCL11	0,20	0,38	6,03E-01	1,00E+00
TOP2B	-0,20	0,38	6,05E-01	1,00E+00
PROC	0,37	0,72	6,07E-01	1,00E+00
ENRAGE	-0,27	0,53	6,09E-01	1,00E+00
CCL11	0,40	0,78	6,10E-01	1,00E+00
RRM2B	-0,29	0,58	6,10E-01	1,00E+00
MCP4	0,27	0,53	6,13E-01	1,00E+00
F7	0,38	0,77	6,18E-01	1,00E+00
SPON2	0,82	1,65	6,18E-01	1,00E+00
CD40L	-0,16	0,33	6,20E-01	1,00E+00
CDH1	0,33	0,66	6,20E-01	1,00E+00
PSMA1	0,23	0,47	6,26E-01	1,00E+00
ST1A1	-0,21	0,43	6,27E-01	1,00E+00
VEGFD	0,41	0,89	6,42E-01	1,00E+00
THPO	-0,43	0,97	6,55E-01	1,00E+00
FCN2	-0,24	0,53	6,56E-01	1,00E+00
SORT1	-0,47	1,05	6,56E-01	1,00E+00
CST5	0,31	0,70	6,58E-01	1,00E+00
CFHR5	0,29	0,67	6,60E-01	1,00E+00
EPO	0,16	0,36	6,60E-01	1,00E+00
ALDH3A1	-0,25	0,59	6,70E-01	1,00E+00
IDUA	0,29	0,70	6,77E-01	1,00E+00
Flt3L	-0,32	0,79	6,80E-01	1,00E+00
LAPTGFbeta1	-0,29	0,72	6,81E-01	1,00E+00
Gal3	0,43	1,05	6,82E-01	1,00E+00
RCOR1	-0,54	1,31	6,83E-01	1,00E+00
MVK	-0,18	0,43	6,83E-01	1,00E+00
SCGB3A2	0,17	0,41	6,83E-01	1,00E+00
VEGFA	0,33	0,82	6,84E-01	1,00E+00
HSP27	-0,55	1,35	6,85E-01	1,00E+00
NRTN	-0,48	1,19	6,86E-01	1,00E+00
KIM1	0,19	0,48	6,88E-01	1,00E+00
PRSS2	0,26	0,65	6,90E-01	1,00E+00
CD84	-0,27	0,68	6,90E-01	1,00E+00
DECR1	-0,13	0,32	6,99E-01	1,00E+00
PAR1	-0,26	0,67	7,03E-01	1,00E+00
MERTK	0,30	0,79	7,04E-01	1,00E+00
SIRT2	-0,11	0,28	7,05E-01	1,00E+00

IL17C	0,22	0,58	7,06E-01	1,00E+00
CCL24	-0,13	0,35	7,12E-01	1,00E+00
SELE	0,20	0,55	7,20E-01	1,00E+00
BLMhydrolase	0,26	0,73	7,22E-01	1,00E+00
CXCL6	0,14	0,39	7,25E-01	1,00E+00
LCN2	1,32	3,84	7,35E-01	1,00E+00
PLXNB2	0,49	1,46	7,39E-01	1,00E+00
Gal4	-0,22	0,68	7,46E-01	1,00E+00
UM	-0,45	1,40	7,49E-01	1,00E+00
HGF	0,30	0,96	7,50E-01	1,00E+00
RARRES2	0,32	1,03	7,55E-01	1,00E+00
CXCL16	0,38	1,21	7,55E-01	1,00E+00
PDP1	0,75	2,41	7,56E-01	1,00E+00
BANK1	-0,10	0,33	7,59E-01	1,00E+00
IL5	0,09	0,29	7,60E-01	1,00E+00
TMPRSS15	-0,44	1,49	7,66E-01	1,00E+00
REN	0,17	0,57	7,68E-01	1,00E+00
CA5A	0,12	0,40	7,70E-01	1,00E+00
MMP12	0,16	0,56	7,73E-01	1,00E+00
CA3	0,46	1,65	7,78E-01	1,00E+00
MCP2	-0,13	0,49	7,84E-01	1,00E+00
XCL1	0,16	0,60	7,86E-01	1,00E+00
MMP1	0,08	0,30	7,87E-01	1,00E+00
FABP4	0,16	0,62	7,93E-01	1,00E+00
CD4	-0,25	0,96	7,96E-01	1,00E+00
RASSF2	-0,21	0,83	8,00E-01	1,00E+00
APOM	0,21	0,84	8,00E-01	1,00E+00
CD40	0,17	0,69	8,02E-01	1,00E+00
STK4	-0,13	0,52	8,04E-01	1,00E+00
Dkk1	-0,14	0,56	8,06E-01	1,00E+00
AMN	0,11	0,46	8,14E-01	1,00E+00
MCP1	0,22	0,98	8,24E-01	1,00E+00
FGF19	0,08	0,36	8,30E-01	1,00E+00
FOXO1	-0,08	0,36	8,36E-01	1,00E+00
CPB1	-0,10	0,48	8,41E-01	1,00E+00
AXIN1	-0,06	0,32	8,43E-01	1,00E+00
ITGAM	0,73	3,63	8,44E-01	1,00E+00
tPA	-0,12	0,61	8,45E-01	1,00E+00
PRSS8	0,18	0,90	8,46E-01	1,00E+00
MEGF9	0,14	0,77	8,56E-01	1,00E+00
CD163	0,12	0,67	8,58E-01	1,00E+00
BTC	0,07	0,37	8,59E-01	1,00E+00
IL4	-0,15	0,87	8,63E-01	1,00E+00
CNTN2	0,12	0,70	8,64E-01	1,00E+00
GRN	0,17	1,02	8,68E-01	1,00E+00
CDCP1	0,10	0,59	8,69E-01	1,00E+00

CHIT1	-0,06	0,39	8,71E-01	1,00E+00
PLA2G7	0,22	1,35	8,72E-01	1,00E+00
HO1	0,14	0,88	8,72E-01	1,00E+00
TIGAR	0,09	0,56	8,76E-01	1,00E+00
CCL18	-0,06	0,36	8,78E-01	1,00E+00
SERPINA5	-0,11	0,75	8,78E-01	1,00E+00
F11	-0,11	0,72	8,79E-01	1,00E+00
PAM	0,13	0,85	8,79E-01	1,00E+00
TNFRSF10A	-0,15	0,98	8,81E-01	1,00E+00
IL15RA	-0,82	5,56	8,83E-01	1,00E+00
CCL17	-0,05	0,35	8,86E-01	1,00E+00
IL33	-0,40	2,96	8,93E-01	1,00E+00
FETUB	-0,07	0,54	9,03E-01	1,00E+00
METAP1	0,24	2,06	9,07E-01	1,00E+00
GLO1	0,05	0,40	9,08E-01	1,00E+00
TCN2	-0,09	0,76	9,10E-01	1,00E+00
GNLY	-0,07	0,76	9,21E-01	1,00E+00
TR	-0,04	0,48	9,28E-01	1,00E+00
ANG1	-0,04	0,46	9,30E-01	1,00E+00
MARCO	0,12	1,45	9,32E-01	1,00E+00
EGFR	-0,13	1,57	9,32E-01	1,00E+00
IL6RA	-0,06	0,79	9,37E-01	1,00E+00
SLAMF7	0,04	0,54	9,37E-01	1,00E+00
CCL4	0,04	0,55	9,46E-01	1,00E+00
FES	-0,06	0,85	9,48E-01	1,00E+00
PI3	0,07	1,18	9,54E-01	1,00E+00
STAMBP	0,02	0,33	9,59E-01	1,00E+00
FGF23	-0,03	0,67	9,59E-01	1,00E+00
APN	0,06	1,11	9,60E-01	1,00E+00
ST6GAL1	0,03	0,59	9,62E-01	1,00E+00
HPGDS	0,03	0,71	9,62E-01	1,00E+00
PVALB	-0,01	0,19	9,64E-01	1,00E+00
CCL16	-0,02	0,60	9,68E-01	1,00E+00
TIMP4	-0,04	0,93	9,69E-01	1,00E+00
MCP3	0,02	0,65	9,72E-01	1,00E+00
PLIN1	-0,02	0,61	9,73E-01	1,00E+00
LEP	-0,01	0,36	9,77E-01	1,00E+00
C2	0,01	0,74	9,88E-01	1,00E+00
CASP8	-0,01	0,50	9,89E-01	1,00E+00
IL2RB	-0,01	1,01	9,95E-01	1,00E+00
NT3	0,00	0,74	9,98E-01	1,00E+00
EGFL7	0,00	0,83	9,98E-01	1,00E+00

Supplemental Table 3: Associations between proteins levels measured at V4 and eGFR slope.

Multivariable linear regression models included including the following variables at V1: age, sex, body mass index, current smoker and eGFR.

Protein at V4	β coefficient	Standard error	P value	Bonferroni P value
PLC	9,87	1,01	1,78E-21	7,97E-19
NPPC	5,09	0,54	4,45E-20	1,99E-17
MB	5,49	0,59	1,16E-19	5,20E-17
IGFBP6	6,73	0,79	3,94E-17	1,77E-14
TNFR1	7,76	0,91	6,25E-17	2,80E-14
MEPE	5,46	0,71	4,08E-14	1,83E-11
PGF	8,90	1,16	4,37E-14	1,96E-11
KLK6	7,38	1,00	2,81E-13	1,26E-10
CST3	5,11	0,69	3,17E-13	1,42E-10
EPHB4	7,99	1,12	1,75E-12	7,84E-10
LTBR	6,88	0,99	6,95E-12	3,11E-09
FABP4	3,19	0,48	6,78E-11	3,04E-08
CA14	6,77	1,04	1,25E-10	5,60E-08
CLEC1A	6,11	0,95	1,60E-10	7,17E-08
TNFR2	5,52	0,85	1,62E-10	7,26E-08
TNFRSF11A	5,07	0,80	3,16E-10	1,42E-07
DLK1	3,15	0,50	3,42E-10	1,53E-07
TFF3	4,43	0,72	1,00E-09	4,48E-07
TNFRSF9	4,57	0,76	2,34E-09	1,05E-06
BAMBI	5,14	0,89	1,13E-08	5,06E-06
ADM	4,51	0,79	1,51E-08	6,76E-06
PI3	5,03	0,90	3,13E-08	1,40E-05
CD93	5,17	1,03	6,53E-07	2,93E-04
COL18A1	3,75	0,76	9,07E-07	4,06E-04
TGFalpha	5,23	1,07	1,09E-06	4,88E-04
MMP3	2,61	0,53	1,16E-06	5,20E-04
PRSS2	2,81	0,58	1,25E-06	5,60E-04
OPN	3,35	0,70	1,71E-06	7,66E-04
CSTB	2,49	0,53	3,44E-06	1,54E-03
TNFRSF14	3,32	0,73	6,82E-06	3,06E-03
FGF23	3,26	0,73	9,17E-06	4,11E-03
CCL15	2,88	0,66	1,34E-05	6,00E-03
CCL14	2,85	0,65	1,42E-05	6,36E-03
TM	4,11	0,94	1,49E-05	6,68E-03
Gal9	4,23	0,97	1,51E-05	6,76E-03
FABP9	3,23	0,75	1,57E-05	7,03E-03
CCL23	2,76	0,64	1,58E-05	7,08E-03

PGLYRP1	2,71	0,63	1,86E-05	8,33E-03
REG1A	2,27	0,54	3,03E-05	1,36E-02
TNFRSF13B	3,43	0,82	3,41E-05	1,53E-02
SCF	3,18	0,77	3,81E-05	1,71E-02
VASN	3,80	0,93	4,49E-05	2,01E-02
AGRP	3,35	0,82	4,77E-05	2,14E-02
IL10RB	4,64	1,14	4,84E-05	2,17E-02
RAGE	3,59	0,88	5,05E-05	2,26E-02
TGFBR3	3,11	0,77	5,25E-05	2,35E-02
IL12B	1,93	0,48	5,51E-05	2,47E-02
IL17C	1,91	0,48	6,75E-05	3,02E-02
MFAP5	3,69	0,93	7,28E-05	3,26E-02
TLT2	2,74	0,70	1,01E-04	4,52E-02
RETN	2,12	0,56	1,76E-04	7,89E-02
ALCAM	4,84	1,29	1,86E-04	8,33E-02
IL18BP	3,60	0,96	1,95E-04	8,73E-02
VSIG2	2,38	0,64	2,00E-04	8,95E-02
Gal3	3,52	0,95	2,11E-04	9,43E-02
DSG4	2,06	0,56	2,72E-04	1,22E-01
REG3A	55,53	2,80	2,81E-04	1,26E-01
CD5	3,20	0,88	3,02E-04	1,35E-01
PRSS27	2,39	0,66	3,26E-04	1,46E-01
CD59	3,44	0,96	3,63E-04	1,62E-01
AXL	3,52	0,99	3,71E-04	1,66E-01
GDF15	2,33	0,66	4,09E-04	1,83E-01
CAPG	1,82	0,51	4,16E-04	1,86E-01
CAPG	1,82	0,51	4,16E-04	1,86E-01
CX3CL1	3,07	0,88	5,13E-04	2,30E-01
TRAILR2	2,32	0,68	6,32E-04	2,83E-01
TF	3,45	1,01	6,64E-04	2,97E-01
PDL1	2,14	0,63	7,92E-04	3,55E-01
CALCA	1,48	0,44	9,02E-04	4,04E-01
IGLC2	1,94	0,59	1,00E-03	4,50E-01
CSF1	4,90	1,49	1,04E-03	4,64E-01
TIMP1	2,31	0,74	1,80E-03	8,04E-01
ANG	1,71	0,55	1,86E-03	8,34E-01
RARRES2	2,97	0,96	1,90E-03	8,52E-01
AGR2	1,09	0,36	2,20E-03	9,86E-01
PLIN1	1,69	0,55	2,23E-03	9,99E-01
MMP10	1,57	0,52	2,33E-03	1,00E+00
GDNF	2,13	0,70	2,45E-03	1,00E+00
MERTK	2,45	0,81	2,49E-03	1,00E+00
PCOLCE	1,94	0,64	2,72E-03	1,00E+00
Gal4	1,82	0,61	2,84E-03	1,00E+00
PTK7	2,85	0,96	3,03E-03	1,00E+00
CST5	1,91	0,64	3,14E-03	1,00E+00

CA12	3,11	1,06	3,38E-03	1,00E+00
FABP2	1,24	0,43	3,94E-03	1,00E+00
TNNI3	1,12	0,39	4,25E-03	1,00E+00
CA4	1,93	0,68	4,40E-03	1,00E+00
IL2RA	2,06	0,73	4,58E-03	1,00E+00
DCN	3,69	1,32	5,26E-03	1,00E+00
CPB1	1,25	0,45	6,00E-03	1,00E+00
CCL16	1,62	0,60	6,49E-03	1,00E+00
CD4	2,56	0,94	6,78E-03	1,00E+00
AMBP	4,23	1,56	6,84E-03	1,00E+00
EFEMP1	2,02	0,75	7,31E-03	1,00E+00
SHPS1	2,05	0,76	7,43E-03	1,00E+00
TR	1,57	0,59	7,44E-03	1,00E+00
UPAR	1,97	0,74	7,54E-03	1,00E+00
CD40	1,79	0,70	1,06E-02	1,00E+00
IL4RA	2,66	1,05	1,17E-02	1,00E+00
NTproBNP	0,91	0,36	1,19E-02	1,00E+00
TNFRSF10A	2,28	0,91	1,30E-02	1,00E+00
IL16	1,35	0,55	1,33E-02	1,00E+00
DNER	-3,39	1,37	1,34E-02	1,00E+00
CA3	2,13	0,86	1,34E-02	1,00E+00
GT	1,34	0,54	1,35E-02	1,00E+00
ARTN	1,69	0,68	1,40E-02	1,00E+00
SPON2	3,73	1,52	1,43E-02	1,00E+00
CDH5	2,38	0,97	1,45E-02	1,00E+00
NUCB2	1,19	0,49	1,47E-02	1,00E+00
LTBP2	-13,96	5,51	1,55E-02	1,00E+00
MMP7	1,89	0,78	1,55E-02	1,00E+00
SPARCL1	2,26	0,93	1,58E-02	1,00E+00
hOSCAR	2,56	1,09	1,86E-02	1,00E+00
FCGR2A	1,16	0,50	1,95E-02	1,00E+00
IL15RA	12,03	5,15	2,03E-02	1,00E+00
IGFBP2	1,09	0,47	2,15E-02	1,00E+00
CD8A	1,05	0,46	2,33E-02	1,00E+00
IGFBP7	2,20	0,97	2,35E-02	1,00E+00
CD46	1,16	0,51	2,35E-02	1,00E+00
FAS	1,84	0,82	2,49E-02	1,00E+00
IDUA	-1,41	0,64	2,72E-02	1,00E+00
ICAM2	1,71	0,77	2,73E-02	1,00E+00
BetaNGF	1,83	0,84	2,89E-02	1,00E+00
TRAP	-1,76	0,81	3,03E-02	1,00E+00
VEGFA	1,73	0,80	3,08E-02	1,00E+00
TNFRSF10C	1,20	0,56	3,13E-02	1,00E+00
CCL18	0,82	0,38	3,20E-02	1,00E+00
LHB	0,77	0,36	3,24E-02	1,00E+00
GIF	0,81	0,38	3,25E-02	1,00E+00

GDF2	-1,84	0,86	3,40E-02	1,00E+00
COL1A1	1,62	0,78	3,87E-02	1,00E+00
PON3	-1,06	0,51	3,92E-02	1,00E+00
CTSD	-1,45	0,70	3,99E-02	1,00E+00
MCP1	1,46	0,72	4,27E-02	1,00E+00
IL1ra	1,03	0,51	4,41E-02	1,00E+00
REN	0,83	0,41	4,48E-02	1,00E+00
PRELP	3,11	1,59	5,09E-02	1,00E+00
CTSL1	1,77	0,91	5,29E-02	1,00E+00
PARP1	0,85	0,44	5,32E-02	1,00E+00
CTSZ	1,61	0,85	5,69E-02	1,00E+00
PIgR	4,13	2,18	5,87E-02	1,00E+00
NCAM1	1,49	0,79	6,08E-02	1,00E+00
FGF19	0,71	0,38	6,49E-02	1,00E+00
SPON1	2,68	1,46	6,69E-02	1,00E+00
TIMP4	1,38	0,75	6,71E-02	1,00E+00
SELP	-0,68	0,37	6,85E-02	1,00E+00
IL18	-1,08	0,60	7,02E-02	1,00E+00
TGM2	0,88	0,49	7,03E-02	1,00E+00
Notch3	1,56	0,90	8,20E-02	1,00E+00
VCAM1	1,43	0,82	8,30E-02	1,00E+00
PDL2	1,55	0,92	8,99E-02	1,00E+00
TFPI	-1,56	0,92	9,18E-02	1,00E+00
UM	-2,17	1,30	9,54E-02	1,00E+00
TMPRSS15	-1,58	0,95	9,81E-02	1,00E+00
CDH1	1,13	0,69	1,03E-01	1,00E+00
IL1RT1	1,77	1,10	1,08E-01	1,00E+00
CES1	-0,65	0,41	1,15E-01	1,00E+00
FES	1,64	1,04	1,16E-01	1,00E+00
MARCO	-2,12	1,38	1,24E-01	1,00E+00
GNLY	0,79	0,51	1,24E-01	1,00E+00
CXCL9	0,64	0,42	1,24E-01	1,00E+00
FCN2	-0,84	0,55	1,27E-01	1,00E+00
CNDP1	-1,04	0,68	1,28E-01	1,00E+00
C2	-1,17	0,78	1,33E-01	1,00E+00
NOTCH1	1,58	1,05	1,33E-01	1,00E+00
NRP1	2,37	1,58	1,34E-01	1,00E+00
PDCD1	1,26	0,84	1,35E-01	1,00E+00
LRP1	-1,06	0,71	1,37E-01	1,00E+00
ACE2	-0,79	0,53	1,38E-01	1,00E+00
IL18	-0,85	0,57	1,40E-01	1,00E+00
CTRC	0,64	0,43	1,40E-01	1,00E+00
LILRB5	0,55	0,38	1,44E-01	1,00E+00
IL17RA	0,76	0,53	1,48E-01	1,00E+00
IGFBP1	0,51	0,35	1,48E-01	1,00E+00
CCL25	0,85	0,59	1,52E-01	1,00E+00

CPA1	0,62	0,43	1,53E-01	1,00E+00
LEP	0,42	0,30	1,58E-01	1,00E+00
CR2	0,81	0,57	1,58E-01	1,00E+00
LILRB1	1,19	0,84	1,60E-01	1,00E+00
PTN	0,64	0,46	1,61E-01	1,00E+00
OPG	-1,31	0,94	1,65E-01	1,00E+00
BNP	1,04	0,75	1,67E-01	1,00E+00
PRCP	3,42	2,40	1,68E-01	1,00E+00
Flt3L	-1,02	0,75	1,72E-01	1,00E+00
PECAM1	-0,53	0,39	1,72E-01	1,00E+00
vWF	0,54	0,41	1,83E-01	1,00E+00
GP1BA	0,49	0,37	1,84E-01	1,00E+00
ERBB2IP	0,64	0,49	1,91E-01	1,00E+00
TSLP	-2,61	1,95	1,93E-01	1,00E+00
ADAMTS13	-2,30	1,79	1,99E-01	1,00E+00
MCP3	0,68	0,54	2,04E-01	1,00E+00
RARRES1	-1,61	1,27	2,06E-01	1,00E+00
APOM	-1,02	0,81	2,09E-01	1,00E+00
AMN	0,52	0,42	2,16E-01	1,00E+00
S2	1,49	1,20	2,17E-01	1,00E+00
TNFB	0,85	0,69	2,20E-01	1,00E+00
LPL	0,81	0,66	2,21E-01	1,00E+00
EGFL7	0,91	0,74	2,22E-01	1,00E+00
PAM	0,99	0,83	2,30E-01	1,00E+00
SERPINA7	-0,93	0,78	2,31E-01	1,00E+00
BLMhydrolase	0,81	0,68	2,33E-01	1,00E+00
TRANCE	0,61	0,51	2,35E-01	1,00E+00
PON2	-0,75	0,64	2,41E-01	1,00E+00
PAI	-0,43	0,37	2,46E-01	1,00E+00
TCN2	-0,84	0,73	2,51E-01	1,00E+00
PTX3	0,73	0,64	2,55E-01	1,00E+00
CEACAM8	0,54	0,48	2,59E-01	1,00E+00
IL17D	1,24	1,10	2,60E-01	1,00E+00
PAR1	0,69	0,63	2,71E-01	1,00E+00
PDGFsubunitB	-0,36	0,33	2,73E-01	1,00E+00
IL2RB	-1,00	0,92	2,76E-01	1,00E+00
CHIT1	0,39	0,36	2,77E-01	1,00E+00
MET	1,09	1,01	2,83E-01	1,00E+00
RRM2B	0,62	0,59	2,89E-01	1,00E+00
ANGPTL3	-0,80	0,76	2,90E-01	1,00E+00
PXN	0,54	0,52	2,96E-01	1,00E+00
CA5A	0,37	0,35	2,97E-01	1,00E+00
TNF	-0,67	0,65	2,98E-01	1,00E+00
MAEA	-0,79	0,76	2,99E-01	1,00E+00
FS	-0,69	0,66	2,99E-01	1,00E+00
LYVE1	0,78	0,76	3,03E-01	1,00E+00

LTA4H	-9,42	6,87	3,04E-01	1,00E+00
IL17A	0,65	0,63	3,04E-01	1,00E+00
IL8	0,41	0,40	3,07E-01	1,00E+00
SORT1	-0,98	0,97	3,12E-01	1,00E+00
CASP8	0,51	0,50	3,13E-01	1,00E+00
PRSS8	0,86	0,86	3,17E-01	1,00E+00
CD244	0,77	0,77	3,20E-01	1,00E+00
HPGDS	0,71	0,72	3,28E-01	1,00E+00
MMP12	0,49	0,50	3,28E-01	1,00E+00
MPO	0,54	0,56	3,31E-01	1,00E+00
CD163	-0,60	0,63	3,41E-01	1,00E+00
SERPINA9	0,51	0,55	3,48E-01	1,00E+00
SERPINA12	0,28	0,30	3,48E-01	1,00E+00
CXCL10	0,33	0,35	3,52E-01	1,00E+00
IL33	-2,92	3,05	3,53E-01	1,00E+00
DPP6	0,83	0,89	3,56E-01	1,00E+00
MMP2	0,92	0,99	3,56E-01	1,00E+00
CD6	0,58	0,63	3,56E-01	1,00E+00
FOXO1	0,34	0,37	3,56E-01	1,00E+00
CA1	0,38	0,41	3,58E-01	1,00E+00
GH	0,15	0,17	3,58E-01	1,00E+00
CNTN2	-0,60	0,66	3,60E-01	1,00E+00
CNTN1	0,93	1,05	3,75E-01	1,00E+00
GLO1	-0,32	0,37	3,82E-01	1,00E+00
GALNT10	-0,94	1,08	3,82E-01	1,00E+00
OSMR	-1,08	1,24	3,83E-01	1,00E+00
SIRT5	0,94	1,08	3,86E-01	1,00E+00
PCSK9	-0,71	0,83	3,91E-01	1,00E+00
PTPRS	1,51	1,76	3,92E-01	1,00E+00
TGFBI	0,61	0,71	3,92E-01	1,00E+00
ENTPD6	-0,93	1,10	3,99E-01	1,00E+00
E4BP1	0,29	0,34	4,00E-01	1,00E+00
HGF	-0,68	0,81	4,03E-01	1,00E+00
TNC	0,50	0,60	4,06E-01	1,00E+00
CDCP1	-0,43	0,52	4,07E-01	1,00E+00
LDLreceptor	-0,44	0,54	4,18E-01	1,00E+00
SRC	-0,28	0,34	4,21E-01	1,00E+00
PSPD	0,35	0,43	4,22E-01	1,00E+00
IL6RA	0,65	0,81	4,24E-01	1,00E+00
ENRAGE	0,36	0,45	4,28E-01	1,00E+00
PRTN3	0,33	0,42	4,32E-01	1,00E+00
FGF21	0,20	0,25	4,33E-01	1,00E+00
NRTN	-0,86	1,09	4,34E-01	1,00E+00
CASP3	-0,17	0,22	4,50E-01	1,00E+00
ADA	0,64	0,85	4,52E-01	1,00E+00
PSGL1	-0,83	1,13	4,60E-01	1,00E+00

PLA2G7	0,97	1,33	4,65E-01	1,00E+00
IL5	0,23	0,32	4,71E-01	1,00E+00
PDGFC	-0,79	1,10	4,71E-01	1,00E+00
PDGFsubunitA	-0,23	0,32	4,73E-01	1,00E+00
IL1alpha	3,13	4,32	4,76E-01	1,00E+00
SCGB3A2	0,28	0,39	4,77E-01	1,00E+00
CXCL16	0,80	1,13	4,78E-01	1,00E+00
PLXNB2	-0,86	1,24	4,87E-01	1,00E+00
APN	0,76	1,10	4,89E-01	1,00E+00
SAA4	-0,37	0,54	4,98E-01	1,00E+00
ICAM3	0,48	0,72	5,04E-01	1,00E+00
LOX1	-0,33	0,50	5,04E-01	1,00E+00
CCL4	-0,31	0,48	5,16E-01	1,00E+00
ST1A1	-0,19	0,29	5,22E-01	1,00E+00
BMP6	0,45	0,70	5,25E-01	1,00E+00
IL10RA	0,30	0,47	5,26E-01	1,00E+00
NOS3	0,49	0,78	5,32E-01	1,00E+00
LILRB2	0,39	0,62	5,32E-01	1,00E+00
EGFR	0,93	1,49	5,32E-01	1,00E+00
ENTPD2	-0,83	1,34	5,35E-01	1,00E+00
IL7	-0,26	0,41	5,38E-01	1,00E+00
HAOX1	-0,13	0,21	5,43E-01	1,00E+00
DECR1	-0,13	0,22	5,44E-01	1,00E+00
IL27	0,47	0,77	5,45E-01	1,00E+00
MVK	-0,21	0,35	5,49E-01	1,00E+00
RASSF2	-0,32	0,53	5,50E-01	1,00E+00
LAT2	0,18	0,30	5,56E-01	1,00E+00
tPA	-0,22	0,37	5,63E-01	1,00E+00
PRKAB1	-0,24	0,41	5,64E-01	1,00E+00
NID1	0,34	0,60	5,65E-01	1,00E+00
AZU1	0,16	0,28	5,70E-01	1,00E+00
F7	0,44	0,78	5,71E-01	1,00E+00
CLSPN	-1,07	1,89	5,72E-01	1,00E+00
CXCL1	0,17	0,30	5,80E-01	1,00E+00
SELE	-0,30	0,54	5,81E-01	1,00E+00
CD40L	-0,14	0,26	5,85E-01	1,00E+00
TIE2	0,66	1,21	5,86E-01	1,00E+00
TNFSF14	-0,30	0,55	5,88E-01	1,00E+00
CXCL5	-0,13	0,25	5,93E-01	1,00E+00
METAP1	1,33	2,54	6,04E-01	1,00E+00
MBL2	0,12	0,24	6,05E-01	1,00E+00
LCN2	-1,22	2,38	6,08E-01	1,00E+00
IL24	0,51	1,01	6,13E-01	1,00E+00
KIT	0,38	0,75	6,15E-01	1,00E+00
CCL5	0,12	0,24	6,15E-01	1,00E+00
ANG1	-0,15	0,30	6,17E-01	1,00E+00

ST2	0,28	0,56	6,18E-01	1,00E+00
AIFM1	0,19	0,38	6,18E-01	1,00E+00
DPP4	0,38	0,78	6,22E-01	1,00E+00
QPCT	0,50	1,02	6,23E-01	1,00E+00
PLXDC1	0,57	1,16	6,25E-01	1,00E+00
CD84	0,30	0,63	6,29E-01	1,00E+00
JAMA	-0,13	0,26	6,30E-01	1,00E+00
LIFR	0,64	1,33	6,32E-01	1,00E+00
IL7R	0,33	0,70	6,34E-01	1,00E+00
KIM1	0,20	0,43	6,36E-01	1,00E+00
PSMA1	-0,21	0,45	6,39E-01	1,00E+00
HSP27	-0,27	0,58	6,43E-01	1,00E+00
NBN	0,19	0,42	6,54E-01	1,00E+00
ST6GAL1	0,26	0,57	6,56E-01	1,00E+00
MAP4K5	-0,08	0,19	6,62E-01	1,00E+00
NEMO	-0,11	0,24	6,64E-01	1,00E+00
OSM	-0,15	0,34	6,68E-01	1,00E+00
Dkk1	0,19	0,46	6,75E-01	1,00E+00
IL4	-0,33	0,80	6,78E-01	1,00E+00
MCP4	0,15	0,36	6,78E-01	1,00E+00
IL18R1	0,32	0,77	6,79E-01	1,00E+00
IL6	0,17	0,41	6,85E-01	1,00E+00
MMP1	0,11	0,27	6,87E-01	1,00E+00
TWEAK	0,42	1,05	6,88E-01	1,00E+00
PPM1B	-0,90	2,18	6,88E-01	1,00E+00
XCL1	0,22	0,56	6,89E-01	1,00E+00
GRN	0,39	0,98	6,90E-01	1,00E+00
FETUB	-0,28	0,71	6,94E-01	1,00E+00
PRKRA	0,20	0,51	6,95E-01	1,00E+00
PTPRJ	0,21	0,54	6,96E-01	1,00E+00
TOP2B	0,15	0,38	6,99E-01	1,00E+00
MAGED1	0,44	1,13	7,01E-01	1,00E+00
IGFBP3	0,28	0,75	7,06E-01	1,00E+00
ITGB1BP2	0,09	0,23	7,07E-01	1,00E+00
ENG	0,37	1,00	7,07E-01	1,00E+00
IL22RA1	-1,07	2,78	7,14E-01	1,00E+00
FGR	-0,15	0,40	7,15E-01	1,00E+00
SELL	0,27	0,75	7,16E-01	1,00E+00
IL13	-0,24	0,65	7,16E-01	1,00E+00
EpCAM	0,10	0,28	7,17E-01	1,00E+00
COMP	0,25	0,68	7,18E-01	1,00E+00
BTC	0,14	0,39	7,23E-01	1,00E+00
CCL19	-0,11	0,30	7,24E-01	1,00E+00
FCGR3B	0,20	0,56	7,24E-01	1,00E+00
uPA	-0,25	0,73	7,31E-01	1,00E+00
IL1RL2	0,25	0,72	7,32E-01	1,00E+00

ADGRG1	-0,15	0,43	7,34E-01	1,00E+00
CFHR5	0,23	0,70	7,38E-01	1,00E+00
THBS2	0,48	1,44	7,40E-01	1,00E+00
BID	0,23	0,75	7,55E-01	1,00E+00
CXCL11	-0,10	0,32	7,63E-01	1,00E+00
NUB1	0,14	0,47	7,68E-01	1,00E+00
IgGFcreceptorIIb	0,11	0,38	7,79E-01	1,00E+00
FOSB	0,15	0,53	7,82E-01	1,00E+00
CES2	-0,29	1,07	7,83E-01	1,00E+00
NCF2	0,08	0,31	7,86E-01	1,00E+00
GP6	0,11	0,41	7,87E-01	1,00E+00
RCOR1	0,23	0,85	7,89E-01	1,00E+00
ENAH	0,20	0,75	7,90E-01	1,00E+00
CCL11	0,18	0,68	7,92E-01	1,00E+00
AOC3	-0,21	0,81	7,94E-01	1,00E+00
ITGAM	0,32	1,22	7,95E-01	1,00E+00
CRH	0,10	0,39	7,97E-01	1,00E+00
FGF5	0,55	2,25	8,06E-01	1,00E+00
SLAMF7	-0,13	0,53	8,08E-01	1,00E+00
CSNK1D	0,22	0,91	8,09E-01	1,00E+00
PROC	0,16	0,69	8,15E-01	1,00E+00
WAS	-0,07	0,29	8,19E-01	1,00E+00
CHI3L1	-0,09	0,38	8,19E-01	1,00E+00
THBS4	-0,12	0,55	8,21E-01	1,00E+00
IL20RA	0,18	0,82	8,22E-01	1,00E+00
STAMBP	-0,07	0,31	8,23E-01	1,00E+00
VASH1	-0,19	0,83	8,24E-01	1,00E+00
MEGF9	0,19	0,84	8,24E-01	1,00E+00
INPPL1	0,05	0,23	8,25E-01	1,00E+00
CCL17	-0,06	0,29	8,28E-01	1,00E+00
FAP	0,18	0,85	8,29E-01	1,00E+00
PAPPA	0,12	0,60	8,38E-01	1,00E+00
TRAIL	-0,23	1,12	8,38E-01	1,00E+00
TIGAR	0,09	0,46	8,41E-01	1,00E+00
CHL1	-0,15	0,81	8,55E-01	1,00E+00
ALDH3A1	0,10	0,53	8,56E-01	1,00E+00
EPO	0,07	0,40	8,60E-01	1,00E+00
IFNgamma	-1,03	5,66	8,61E-01	1,00E+00
VEGFC	-0,08	0,46	8,64E-01	1,00E+00
BANK1	-0,04	0,22	8,67E-01	1,00E+00
STX8	-0,07	0,41	8,69E-01	1,00E+00
PDP1	0,57	3,43	8,71E-01	1,00E+00
LIF	-0,13	0,81	8,71E-01	1,00E+00
IL1RT2	-0,16	0,97	8,72E-01	1,00E+00
GAS6	0,12	0,78	8,72E-01	1,00E+00
IL10	0,10	0,62	8,74E-01	1,00E+00

SERPINA5	0,10	0,74	8,89E-01	1,00E+00
ATP6AP2	0,16	1,23	8,96E-01	1,00E+00
MCP2	0,06	0,47	8,97E-01	1,00E+00
NT3	0,09	0,66	8,97E-01	1,00E+00
THPO	0,11	0,90	9,00E-01	1,00E+00
PLTP	-0,12	0,93	9,00E-01	1,00E+00
ST3GAL1	0,07	0,56	9,02E-01	1,00E+00
CALR	0,14	1,15	9,04E-01	1,00E+00
IL20	-0,12	0,99	9,04E-01	1,00E+00
SIRT2	-0,03	0,27	9,05E-01	1,00E+00
SLAMF1	-0,08	0,66	9,06E-01	1,00E+00
VEGFD	0,09	0,76	9,08E-01	1,00E+00
SMAD1	-0,05	0,45	9,11E-01	1,00E+00
F11	-0,08	0,77	9,16E-01	1,00E+00
C1QTNF1	-0,05	0,48	9,19E-01	1,00E+00
PVALB	0,02	0,18	9,29E-01	1,00E+00
TIE1	-0,10	1,25	9,34E-01	1,00E+00
CRTAC1	-0,04	0,49	9,34E-01	1,00E+00
S1	0,17	2,06	9,36E-01	1,00E+00
CCL24	-0,03	0,34	9,38E-01	1,00E+00
MAX	-0,04	0,47	9,38E-01	1,00E+00
ITGB2	0,04	0,60	9,41E-01	1,00E+00
LAPTGFbeta1	-0,06	0,76	9,41E-01	1,00E+00
ICAM1	0,06	0,77	9,42E-01	1,00E+00
TIMD4	-0,04	0,60	9,42E-01	1,00E+00
STK4	-0,02	0,28	9,44E-01	1,00E+00
TNXB	-0,10	1,53	9,46E-01	1,00E+00
HBEGF	0,03	0,40	9,49E-01	1,00E+00
MMP9	-0,02	0,36	9,50E-01	1,00E+00
AXIN1	-0,01	0,24	9,59E-01	1,00E+00
CCL28	-0,04	0,70	9,59E-01	1,00E+00
CCL20	0,01	0,32	9,63E-01	1,00E+00
STXBP3	0,04	0,80	9,64E-01	1,00E+00
FKBP1B	-0,03	0,58	9,64E-01	1,00E+00
BOC	-0,05	1,13	9,66E-01	1,00E+00
RASA1	-0,03	0,80	9,69E-01	1,00E+00
CXCL6	0,01	0,32	9,86E-01	1,00E+00
YES1	0,00	0,22	9,86E-01	1,00E+00
EDIL3	-0,02	1,79	9,89E-01	1,00E+00
TNFSF13B	0,00	0,96	9,97E-01	1,00E+00
DEFA1	-0,01	1,54	9,97E-01	1,00E+00
ITGB1BP1	-0,01	1,78	9,98E-01	1,00E+00
HO1	0,00	0,81	9,99E-01	1,00E+00

Supplemental Table 4: Associations between genes expressions measured at V4 and eGFR slope.

Multivariable linear regression models included including the following variables at V1: age, sex, body mass index, current smoker and eGFR.

Gene at V4	β coefficient	Standard error	P value	Bonferroni P value
RPL36AP51	-1,93	0,59	0,001	0,034
THBS1	-1,24	0,39	0,002	0,07
CCL18	3,30	1,11	0,003	0,10
LINC00681	-2,47	0,86	0,004	0,14
LOC102724434_AC073130.1	2,43	0,88	0,006	0,20
RP11_494K3.2	-2,98	1,10	0,007	0,24
SESN3	-1,15	0,43	0,007	0,24
RP11_266O8.1	3,30	1,23	0,008	0,27
LYRM5	-1,53	0,58	0,009	0,31
NEIL3	-1,86	0,74	0,012	0,41
CYP46A1	2,40	0,97	0,014	0,48
DNAJC6	-0,71	0,34	0,037	1
VEGFB	1,97	0,95	0,038	1
RP11_736K20.4_RP11_736K20.5	-2,07	1,02	0,044	1
RPL21P10	-1,24	0,65	0,06	1
VEGFC	1,24	0,67	0,07	1
KLK6	2,15	1,35	0,11	1
CXADRP2	1,43	0,96	0,14	1
MIR1205	0,84	0,60	0,16	1
LYRM5.1	-0,64	0,50	0,20	1
ICAM1	1,22	0,97	0,21	1
FLT1	1,05	0,91	0,25	1
OR5M4P	0,83	0,77	0,28	1
SESN3.1	-0,31	0,29	0,30	1
PIGF	-0,54	0,53	0,31	1
F3	-1,24	1,27	0,33	1
MMP2	0,90	0,96	0,35	1
TIMP4	-0,82	0,93	0,38	1
FGF23	0,98	1,59	0,54	1
VEGFA	0,30	0,58	0,60	1
TGFA	-0,31	0,76	0,68	1
TIMP1	-0,18	0,47	0,70	1
PGF	0,19	1,25	0,88	1
VCAM1	0,06	0,80	0,94	1

Supplemental Table 5: Associations between proteins levels measured at V1 and ΔeGFR≥15mL/min/1.7m².

Multivariable logistic regression models included including the following variables at V1: age, sex, body mass index, current smoker and eGFR.

Protein at V1	β coefficient	Standard error	P value	Bonferroni P value
PAPPA	0,07	0,02	1,56E-04	6,95E-02
PDL1	0,08	0,02	4,00E-04	1,78E-01
TNFRSF9	0,10	0,03	5,65E-04	2,52E-01
GDNF	0,08	0,03	1,73E-03	7,70E-01
VCAM1	0,09	0,03	1,90E-03	8,49E-01
CCL19	0,04	0,01	2,03E-03	9,04E-01
PXN	0,08	0,03	2,16E-03	9,65E-01
CST3	0,08	0,03	2,52E-03	1,00E+00
NPPC	0,06	0,02	2,77E-03	1,00E+00
TRAP	-0,09	0,03	3,02E-03	1,00E+00
NCF2	0,06	0,02	3,32E-03	1,00E+00
COL1A1	0,08	0,03	4,66E-03	1,00E+00
MBL2	0,02	0,01	5,62E-03	1,00E+00
NID1	0,06	0,02	6,66E-03	1,00E+00
TGFalpha	0,09	0,03	6,85E-03	1,00E+00
PGLYRP1	0,07	0,03	1,17E-02	1,00E+00
GP1BA	0,03	0,01	1,25E-02	1,00E+00
CD46	0,04	0,02	1,28E-02	1,00E+00
DCN	0,13	0,05	1,38E-02	1,00E+00
Gal9	0,09	0,04	1,47E-02	1,00E+00
NOS3	0,06	0,02	1,54E-02	1,00E+00
THBS2	0,13	0,05	1,56E-02	1,00E+00
LHB	0,03	0,01	1,61E-02	1,00E+00
CAPG	0,05	0,02	1,71E-02	1,00E+00
CAPG	0,05	0,02	1,71E-02	1,00E+00
IL16	0,06	0,03	1,73E-02	1,00E+00
RASA1	0,06	0,02	1,79E-02	1,00E+00
BetaNGF	0,09	0,04	2,00E-02	1,00E+00
ENG	0,08	0,04	2,02E-02	1,00E+00
PON3	-0,04	0,02	2,10E-02	1,00E+00
OPN	0,06	0,03	2,16E-02	1,00E+00
TNFRSF10C	0,04	0,02	2,32E-02	1,00E+00
GDF15	0,05	0,02	2,47E-02	1,00E+00
VEGFC	0,04	0,02	2,80E-02	1,00E+00
IGFBP6	0,06	0,03	2,95E-02	1,00E+00
TIMP1	0,06	0,03	3,04E-02	1,00E+00
IL1ra	0,04	0,02	3,20E-02	1,00E+00

MEPE	0,06	0,03	3,39E-02	1,00E+00
CCL14	0,05	0,02	3,40E-02	1,00E+00
uPA	-0,06	0,03	3,46E-02	1,00E+00
ENTPD6	-0,08	0,04	3,57E-02	1,00E+00
ST3GAL1	-0,04	0,02	3,61E-02	1,00E+00
PIgR	0,18	0,09	3,64E-02	1,00E+00
hOSCAR	0,09	0,04	3,78E-02	1,00E+00
LPL	-0,03	0,02	3,86E-02	1,00E+00
TGFBI	0,05	0,02	3,87E-02	1,00E+00
VASN	0,07	0,03	4,06E-02	1,00E+00
ICAM1	0,06	0,03	4,18E-02	1,00E+00
ITGB1BP1	-0,09	0,05	4,27E-02	1,00E+00
COL18A1	0,06	0,03	4,41E-02	1,00E+00
LILRB1	0,06	0,03	4,43E-02	1,00E+00
CA4	0,07	0,04	4,48E-02	1,00E+00
LILRB5	0,03	0,01	4,66E-02	1,00E+00
LTA4H	0,66	0,25	4,79E-02	1,00E+00
PGF	0,08	0,04	5,07E-02	1,00E+00
PDCD1	0,05	0,03	5,20E-02	1,00E+00
PCOLCE	0,05	0,02	5,30E-02	1,00E+00
NOTCH1	0,07	0,04	5,71E-02	1,00E+00
SERPINA9	0,04	0,02	6,13E-02	1,00E+00
CD5	0,06	0,04	6,57E-02	1,00E+00
TNFR2	0,06	0,03	6,76E-02	1,00E+00
TNFRSF13B	0,06	0,03	6,88E-02	1,00E+00
MMP10	0,03	0,02	7,02E-02	1,00E+00
IL27	0,05	0,03	7,23E-02	1,00E+00
TSLP	0,07	0,04	7,45E-02	1,00E+00
OSM	0,02	0,01	7,51E-02	1,00E+00
MCP1	-0,06	0,03	7,62E-02	1,00E+00
SCF	0,05	0,03	7,72E-02	1,00E+00
CCL25	-0,03	0,02	7,75E-02	1,00E+00
CCL5	0,02	0,01	8,03E-02	1,00E+00
TGFBR3	0,05	0,03	8,03E-02	1,00E+00
FGR	0,03	0,02	8,14E-02	1,00E+00
MET	0,06	0,04	8,23E-02	1,00E+00
TM	0,07	0,04	8,34E-02	1,00E+00
CD6	0,04	0,02	8,38E-02	1,00E+00
PRCP	-0,26	0,12	8,41E-02	1,00E+00
IL10RB	0,07	0,04	8,56E-02	1,00E+00
CSF1	0,08	0,05	8,72E-02	1,00E+00
IGFBP3	0,05	0,03	8,81E-02	1,00E+00
TNFSF14	0,03	0,02	9,38E-02	1,00E+00
SERPINA12	0,02	0,01	9,60E-02	1,00E+00
LOX1	0,03	0,02	1,01E-01	1,00E+00
SPARCL1	0,05	0,03	1,04E-01	1,00E+00

PARP1	0,04	0,02	1,05E-01	1,00E+00
PLC	0,06	0,04	1,06E-01	1,00E+00
RETN	0,04	0,02	1,07E-01	1,00E+00
CCL23	0,04	0,02	1,09E-01	1,00E+00
MERTK	0,04	0,03	1,11E-01	1,00E+00
MAGED1	-0,05	0,03	1,11E-01	1,00E+00
CPA1	-0,02	0,02	1,18E-01	1,00E+00
LILRB2	0,04	0,03	1,19E-01	1,00E+00
RCOR1	-0,07	0,04	1,22E-01	1,00E+00
MFAP5	0,05	0,03	1,23E-01	1,00E+00
PRSS27	0,04	0,02	1,30E-01	1,00E+00
ADGRG1	0,05	0,03	1,31E-01	1,00E+00
IGLC2	0,03	0,02	1,32E-01	1,00E+00
EDIL3	0,12	0,08	1,32E-01	1,00E+00
BMP6	0,04	0,02	1,35E-01	1,00E+00
TNNI3	-0,03	0,02	1,36E-01	1,00E+00
UPAR	0,05	0,03	1,38E-01	1,00E+00
ICAM3	0,05	0,03	1,39E-01	1,00E+00
SELL	0,04	0,03	1,40E-01	1,00E+00
REN	0,03	0,02	1,43E-01	1,00E+00
TNFB	0,04	0,02	1,44E-01	1,00E+00
CD93	0,05	0,04	1,52E-01	1,00E+00
MMP9	0,02	0,02	1,59E-01	1,00E+00
CLEC1A	0,05	0,03	1,68E-01	1,00E+00
ANG	0,02	0,02	1,69E-01	1,00E+00
NCAM1	0,04	0,03	1,70E-01	1,00E+00
CFHR5	0,03	0,02	1,70E-01	1,00E+00
TNFR1	0,05	0,04	1,72E-01	1,00E+00
PPM1B	-0,15	0,10	1,72E-01	1,00E+00
PLXNB2	0,07	0,05	1,75E-01	1,00E+00
FKBP1B	-0,02	0,01	1,75E-01	1,00E+00
CEACAM8	0,03	0,02	1,78E-01	1,00E+00
TRAILR2	0,03	0,02	1,80E-01	1,00E+00
TRAIL	0,05	0,03	1,80E-01	1,00E+00
MARCO	0,07	0,05	1,83E-01	1,00E+00
CXCL5	0,02	0,01	1,84E-01	1,00E+00
vWF	0,02	0,02	1,86E-01	1,00E+00
IL18R1	0,04	0,03	1,87E-01	1,00E+00
TNFRSF11A	0,04	0,03	1,89E-01	1,00E+00
DLK1	0,02	0,02	1,90E-01	1,00E+00
CD4	0,04	0,03	1,90E-01	1,00E+00
IL12B	0,02	0,02	1,95E-01	1,00E+00
MPO	0,03	0,03	1,97E-01	1,00E+00
IgGFcreceptorIIb	-0,02	0,01	1,97E-01	1,00E+00
PTX3	0,03	0,02	2,00E-01	1,00E+00
S2	0,06	0,05	2,04E-01	1,00E+00

UM	-0,06	0,05	2,04E-01	1,00E+00
PON2	-0,03	0,02	2,04E-01	1,00E+00
TLT2	0,04	0,03	2,05E-01	1,00E+00
PSGL1	0,05	0,04	2,05E-01	1,00E+00
ADA	0,04	0,03	2,06E-01	1,00E+00
GLO1	0,02	0,01	2,15E-01	1,00E+00
IL8	-0,02	0,02	2,19E-01	1,00E+00
FGF21	-0,01	0,01	2,19E-01	1,00E+00
CR2	0,02	0,02	2,22E-01	1,00E+00
CES1	0,02	0,02	2,25E-01	1,00E+00
STXBP3	-0,02	0,02	2,25E-01	1,00E+00
FCGR3B	0,02	0,02	2,27E-01	1,00E+00
LTBR	0,04	0,04	2,33E-01	1,00E+00
EPHB4	0,05	0,04	2,35E-01	1,00E+00
CTSL1	0,04	0,04	2,42E-01	1,00E+00
IL4RA	0,04	0,04	2,45E-01	1,00E+00
AOC3	0,03	0,03	2,46E-01	1,00E+00
NBN	0,03	0,02	2,47E-01	1,00E+00
CSTB	0,03	0,02	2,50E-01	1,00E+00
IL10	0,02	0,02	2,50E-01	1,00E+00
S1	0,08	0,07	2,55E-01	1,00E+00
APN	-0,04	0,04	2,56E-01	1,00E+00
PRTN3	0,03	0,02	2,56E-01	1,00E+00
CNTN2	-0,03	0,02	2,57E-01	1,00E+00
IL2RA	0,03	0,03	2,58E-01	1,00E+00
TFPI	-0,04	0,03	2,61E-01	1,00E+00
ANG1	0,02	0,02	2,62E-01	1,00E+00
LRP1	-0,03	0,03	2,62E-01	1,00E+00
IL24	-0,03	0,03	2,71E-01	1,00E+00
CDCP1	0,02	0,02	2,73E-01	1,00E+00
CA12	0,04	0,03	2,76E-01	1,00E+00
SORT1	0,04	0,04	2,78E-01	1,00E+00
EFEMP1	0,03	0,03	2,80E-01	1,00E+00
NRP1	0,07	0,06	2,80E-01	1,00E+00
PLTP	0,04	0,03	2,87E-01	1,00E+00
TWEAK	0,04	0,04	2,89E-01	1,00E+00
PDL2	0,03	0,03	2,90E-01	1,00E+00
CSNK1D	0,03	0,02	2,93E-01	1,00E+00
AMBP	0,06	0,06	2,94E-01	1,00E+00
CES2	0,06	0,06	2,96E-01	1,00E+00
CD84	0,02	0,02	2,96E-01	1,00E+00
MAEA	-0,02	0,02	2,96E-01	1,00E+00
TIGAR	0,02	0,02	2,96E-01	1,00E+00
FOXO1	0,01	0,01	2,97E-01	1,00E+00
SLAMF1	0,03	0,03	2,99E-01	1,00E+00
IL17C	-0,02	0,02	3,01E-01	1,00E+00

CRH	0,01	0,01	3,02E-01	1,00E+00
MCP3	0,02	0,02	3,04E-01	1,00E+00
IL17RA	0,02	0,02	3,04E-01	1,00E+00
CPB1	-0,02	0,02	3,04E-01	1,00E+00
IL7	0,02	0,02	3,08E-01	1,00E+00
EGFR	-0,05	0,05	3,10E-01	1,00E+00
MMP12	0,02	0,02	3,10E-01	1,00E+00
ALCAM	0,05	0,05	3,14E-01	1,00E+00
BID	-0,03	0,03	3,14E-01	1,00E+00
ATP6AP2	0,04	0,04	3,17E-01	1,00E+00
FGF19	-0,01	0,01	3,18E-01	1,00E+00
AMN	0,02	0,02	3,21E-01	1,00E+00
IL20	-0,04	0,04	3,22E-01	1,00E+00
CTSD	-0,03	0,03	3,22E-01	1,00E+00
ALDH3A1	-0,02	0,02	3,27E-01	1,00E+00
SAA4	0,02	0,02	3,29E-01	1,00E+00
ST6GAL1	0,02	0,02	3,29E-01	1,00E+00
FABP9	0,02	0,03	3,34E-01	1,00E+00
IL1RT2	0,03	0,04	3,34E-01	1,00E+00
IL15RA	-0,19	0,19	3,36E-01	1,00E+00
FAS	-0,03	0,03	3,37E-01	1,00E+00
SELE	-0,02	0,02	3,39E-01	1,00E+00
TIMD4	0,02	0,02	3,43E-01	1,00E+00
RARRES1	-0,04	0,05	3,44E-01	1,00E+00
PTN	0,02	0,02	3,45E-01	1,00E+00
SIRT5	0,04	0,04	3,49E-01	1,00E+00
SLAMF7	-0,02	0,02	3,51E-01	1,00E+00
FETUB	0,02	0,02	3,52E-01	1,00E+00
METAP1	0,06	0,06	3,55E-01	1,00E+00
RASSF2	0,03	0,03	3,55E-01	1,00E+00
FOSB	0,02	0,02	3,55E-01	1,00E+00
CXCL1	0,02	0,02	3,59E-01	1,00E+00
EGFL7	-0,03	0,03	3,60E-01	1,00E+00
TNFRSF10A	0,03	0,03	3,65E-01	1,00E+00
PTPRS	0,07	0,07	3,65E-01	1,00E+00
TIE1	0,04	0,05	3,65E-01	1,00E+00
NTproBNP	0,01	0,02	3,67E-01	1,00E+00
TNFRSF14	0,02	0,03	3,73E-01	1,00E+00
F7	0,02	0,03	3,74E-01	1,00E+00
SERPINA7	0,02	0,02	3,74E-01	1,00E+00
MMP7	-0,02	0,02	3,75E-01	1,00E+00
Dkk1	0,02	0,02	3,76E-01	1,00E+00
OSMR	0,04	0,05	3,76E-01	1,00E+00
CASP3	-0,01	0,01	3,76E-01	1,00E+00
FGF5	0,06	0,07	3,80E-01	1,00E+00
CALR	-0,04	0,05	3,85E-01	1,00E+00

Notch3	0,03	0,03	3,85E-01	1,00E+00
CCL16	-0,02	0,02	3,86E-01	1,00E+00
CXCL10	0,01	0,01	3,88E-01	1,00E+00
PDGFsubunitB	0,02	0,02	4,05E-01	1,00E+00
PECAM1	-0,01	0,01	4,09E-01	1,00E+00
CD244	0,02	0,03	4,10E-01	1,00E+00
IL18	-0,02	0,02	4,16E-01	1,00E+00
DEFA1	-0,22	0,25	4,24E-01	1,00E+00
TMPRSS15	-0,04	0,05	4,26E-01	1,00E+00
CHI3L1	-0,01	0,02	4,27E-01	1,00E+00
CASP8	0,01	0,02	4,30E-01	1,00E+00
BTC	0,01	0,01	4,31E-01	1,00E+00
TNXB	0,04	0,05	4,38E-01	1,00E+00
TNFSF13B	0,03	0,03	4,39E-01	1,00E+00
KIM1	0,01	0,02	4,40E-01	1,00E+00
VASH1	-0,01	0,01	4,41E-01	1,00E+00
C1QTNF1	0,02	0,02	4,49E-01	1,00E+00
QPCT	0,04	0,05	4,49E-01	1,00E+00
TNC	0,02	0,02	4,49E-01	1,00E+00
CCL17	0,01	0,01	4,51E-01	1,00E+00
PROC	0,02	0,02	4,52E-01	1,00E+00
TGM2	-0,01	0,02	4,57E-01	1,00E+00
GRN	-0,03	0,03	4,64E-01	1,00E+00
RAGE	0,02	0,03	4,64E-01	1,00E+00
Flt3L	-0,02	0,03	4,67E-01	1,00E+00
CD40L	0,01	0,01	4,74E-01	1,00E+00
CCL28	0,02	0,03	4,75E-01	1,00E+00
CNDP1	0,02	0,02	4,80E-01	1,00E+00
ARTN	-0,02	0,02	4,86E-01	1,00E+00
PTPRJ	0,01	0,01	4,86E-01	1,00E+00
FABP2	0,01	0,01	4,90E-01	1,00E+00
PTK7	0,02	0,03	4,90E-01	1,00E+00
PRSS8	0,02	0,03	4,91E-01	1,00E+00
XCL1	0,01	0,02	4,91E-01	1,00E+00
WAS	-0,01	0,01	4,92E-01	1,00E+00
CST5	0,02	0,02	4,94E-01	1,00E+00
PSPD	0,01	0,01	4,99E-01	1,00E+00
ANGPTL3	0,02	0,02	5,02E-01	1,00E+00
PDGFC	-0,02	0,03	5,02E-01	1,00E+00
IGFBP1	0,01	0,01	5,06E-01	1,00E+00
MCP4	0,01	0,02	5,07E-01	1,00E+00
CCL24	-0,01	0,01	5,13E-01	1,00E+00
IL17D	0,03	0,04	5,13E-01	1,00E+00
ENTPD2	-0,04	0,06	5,14E-01	1,00E+00
FCN2	0,01	0,02	5,14E-01	1,00E+00
SPON2	0,04	0,06	5,15E-01	1,00E+00

IL18	-0,01	0,02	5,22E-01	1,00E+00
HPGDS	-0,02	0,02	5,25E-01	1,00E+00
SERPINA5	0,02	0,03	5,26E-01	1,00E+00
BNP	0,05	0,07	5,27E-01	1,00E+00
tPA	-0,01	0,02	5,28E-01	1,00E+00
JAMA	-0,01	0,01	5,31E-01	1,00E+00
KIT	0,02	0,03	5,40E-01	1,00E+00
CXCL16	-0,02	0,04	5,41E-01	1,00E+00
HGF	0,02	0,03	5,45E-01	1,00E+00
AGR2	-0,01	0,02	5,46E-01	1,00E+00
TNF	0,01	0,02	5,46E-01	1,00E+00
IDUA	0,01	0,02	5,47E-01	1,00E+00
IL18BP	0,02	0,03	5,48E-01	1,00E+00
STX8	0,01	0,02	5,50E-01	1,00E+00
SIRT2	0,01	0,01	5,55E-01	1,00E+00
LAT2	0,01	0,01	5,56E-01	1,00E+00
FCGR2A	0,01	0,02	5,57E-01	1,00E+00
NRTN	-0,02	0,04	5,59E-01	1,00E+00
LCN2	0,08	0,13	5,60E-01	1,00E+00
CX3CL1	0,02	0,03	5,64E-01	1,00E+00
IL2RB	-0,02	0,03	5,64E-01	1,00E+00
ITGB1BP2	0,01	0,01	5,66E-01	1,00E+00
TOP2B	0,01	0,01	5,71E-01	1,00E+00
IL33	-0,08	0,14	5,72E-01	1,00E+00
CD40	0,01	0,02	5,75E-01	1,00E+00
IL17A	-0,01	0,02	5,76E-01	1,00E+00
PI3	0,02	0,04	5,80E-01	1,00E+00
PLXDC1	0,02	0,04	5,85E-01	1,00E+00
TRANCE	0,01	0,02	5,86E-01	1,00E+00
IGFBP7	-0,02	0,03	5,87E-01	1,00E+00
CCL20	-0,01	0,01	5,91E-01	1,00E+00
CXCL11	-0,01	0,01	5,94E-01	1,00E+00
C2	0,01	0,03	5,96E-01	1,00E+00
IL5	0,00	0,01	6,00E-01	1,00E+00
MMP3	0,01	0,02	6,02E-01	1,00E+00
DNER	-0,02	0,05	6,04E-01	1,00E+00
GDF2	0,02	0,03	6,12E-01	1,00E+00
PLA2G7	0,02	0,05	6,18E-01	1,00E+00
BLMhydrolase	0,01	0,02	6,19E-01	1,00E+00
MB	-0,01	0,02	6,21E-01	1,00E+00
TIE2	0,02	0,05	6,22E-01	1,00E+00
GIF	-0,01	0,01	6,23E-01	1,00E+00
PRKRA	-0,01	0,02	6,23E-01	1,00E+00
IL22RA1	-0,08	0,16	6,24E-01	1,00E+00
PSMA1	0,01	0,02	6,25E-01	1,00E+00
STAMBP	0,01	0,01	6,25E-01	1,00E+00

PAM	0,01	0,03	6,29E-01	1,00E+00
CLSPN	-0,04	0,08	6,33E-01	1,00E+00
NUCB2	0,01	0,01	6,34E-01	1,00E+00
IL6	0,01	0,01	6,38E-01	1,00E+00
EPO	-0,01	0,01	6,43E-01	1,00E+00
VSIG2	-0,01	0,03	6,44E-01	1,00E+00
AGRP	-0,01	0,03	6,48E-01	1,00E+00
CCL4	-0,01	0,02	6,55E-01	1,00E+00
ICAM2	-0,01	0,03	6,57E-01	1,00E+00
HAOX1	0,00	0,01	6,58E-01	1,00E+00
PDP1	0,04	0,08	6,61E-01	1,00E+00
KLK6	0,01	0,03	6,66E-01	1,00E+00
STK4	0,01	0,02	6,66E-01	1,00E+00
IL10RA	-0,01	0,02	6,70E-01	1,00E+00
TF	0,02	0,04	6,76E-01	1,00E+00
ADAMTS13	-0,03	0,07	6,81E-01	1,00E+00
MVK	0,01	0,01	6,84E-01	1,00E+00
ITGAM	0,07	0,17	6,84E-01	1,00E+00
NT3	-0,01	0,02	6,85E-01	1,00E+00
TIMP4	-0,01	0,03	6,86E-01	1,00E+00
IL4	-0,01	0,03	6,86E-01	1,00E+00
GAS6	0,01	0,03	6,97E-01	1,00E+00
E4BP1	0,00	0,01	6,99E-01	1,00E+00
LDLreceptor	0,01	0,02	7,01E-01	1,00E+00
INPPL1	0,00	0,01	7,03E-01	1,00E+00
SELP	-0,01	0,01	7,04E-01	1,00E+00
ACE2	-0,01	0,02	7,05E-01	1,00E+00
MEGF9	0,01	0,03	7,15E-01	1,00E+00
CXCL9	0,00	0,01	7,16E-01	1,00E+00
SMAD1	0,00	0,01	7,23E-01	1,00E+00
LYVE1	0,01	0,03	7,28E-01	1,00E+00
LAPTGFbeta1	0,01	0,02	7,28E-01	1,00E+00
TFF3	0,00	0,01	7,29E-01	1,00E+00
COMP	-0,01	0,02	7,31E-01	1,00E+00
EpCAM	0,00	0,01	7,32E-01	1,00E+00
Gal3	0,01	0,04	7,41E-01	1,00E+00
CALCA	0,01	0,02	7,43E-01	1,00E+00
PAR1	0,01	0,02	7,44E-01	1,00E+00
REG1A	0,01	0,02	7,46E-01	1,00E+00
CA3	0,02	0,05	7,48E-01	1,00E+00
TCN2	0,01	0,03	7,51E-01	1,00E+00
CRTAC1	-0,01	0,02	7,52E-01	1,00E+00
FS	0,01	0,02	7,52E-01	1,00E+00
APOM	0,01	0,03	7,53E-01	1,00E+00
CCL18	0,00	0,01	7,55E-01	1,00E+00
CD59	0,02	0,06	7,56E-01	1,00E+00

CHL1	0,01	0,03	7,58E-01	1,00E+00
RARRES2	0,01	0,03	7,58E-01	1,00E+00
BOC	0,01	0,04	7,59E-01	1,00E+00
FAP	-0,01	0,03	7,62E-01	1,00E+00
F11	0,01	0,02	7,63E-01	1,00E+00
SRC	0,01	0,04	7,70E-01	1,00E+00
GH	0,00	0,01	7,71E-01	1,00E+00
ITGB2	0,01	0,03	7,71E-01	1,00E+00
CTRC	0,00	0,02	7,75E-01	1,00E+00
ADM	0,01	0,03	7,78E-01	1,00E+00
IL7R	0,01	0,03	7,80E-01	1,00E+00
DSG4	0,01	0,02	7,82E-01	1,00E+00
IL1alpha	0,01	0,05	7,90E-01	1,00E+00
YES1	0,00	0,01	7,93E-01	1,00E+00
THBS4	0,01	0,02	7,96E-01	1,00E+00
NUB1	0,00	0,01	7,98E-01	1,00E+00
CD8A	0,00	0,02	7,99E-01	1,00E+00
GALNT10	-0,01	0,04	8,02E-01	1,00E+00
HBEGF	0,00	0,01	8,06E-01	1,00E+00
Gal4	-0,01	0,02	8,11E-01	1,00E+00
CDH1	0,01	0,02	8,14E-01	1,00E+00
MMP1	0,00	0,01	8,17E-01	1,00E+00
SPON1	-0,01	0,06	8,18E-01	1,00E+00
LIFR	0,01	0,04	8,24E-01	1,00E+00
CA14	0,01	0,04	8,27E-01	1,00E+00
LEP	0,00	0,01	8,29E-01	1,00E+00
AXL	0,01	0,03	8,30E-01	1,00E+00
IL1RT1	0,01	0,04	8,30E-01	1,00E+00
ENRAGE	0,00	0,02	8,30E-01	1,00E+00
PDGFsubunitA	0,00	0,02	8,33E-01	1,00E+00
TR	0,00	0,02	8,33E-01	1,00E+00
CHIT1	0,00	0,01	8,36E-01	1,00E+00
OPG	-0,01	0,03	8,37E-01	1,00E+00
IFNgamma	-0,07	0,33	8,38E-01	1,00E+00
FABP4	0,00	0,02	8,47E-01	1,00E+00
BAMBI	0,01	0,03	8,48E-01	1,00E+00
ERBB2IP	0,00	0,01	8,55E-01	1,00E+00
CCL15	0,00	0,02	8,57E-01	1,00E+00
HSP27	-0,01	0,05	8,58E-01	1,00E+00
MMP2	0,01	0,03	8,60E-01	1,00E+00
ST1A1	0,00	0,01	8,68E-01	1,00E+00
FES	0,00	0,03	8,69E-01	1,00E+00
FGF23	0,00	0,02	8,70E-01	1,00E+00
AXIN1	0,00	0,01	8,74E-01	1,00E+00
GNLY	0,00	0,03	8,77E-01	1,00E+00
PRKAB1	0,00	0,01	8,78E-01	1,00E+00

IGFBP2	0,00	0,02	8,89E-01	1,00E+00
GT	0,00	0,02	8,94E-01	1,00E+00
CDH5	0,00	0,03	8,96E-01	1,00E+00
IL13	0,00	0,02	9,01E-01	1,00E+00
VEGFA	0,00	0,03	9,03E-01	1,00E+00
DPP4	0,00	0,03	9,04E-01	1,00E+00
CTSZ	0,00	0,03	9,08E-01	1,00E+00
MCP2	0,00	0,02	9,11E-01	1,00E+00
VEGFD	0,00	0,03	9,12E-01	1,00E+00
CNTN1	0,00	0,04	9,16E-01	1,00E+00
MAX	0,00	0,01	9,16E-01	1,00E+00
LIF	0,00	0,03	9,25E-01	1,00E+00
GP6	0,00	0,02	9,29E-01	1,00E+00
DPP6	0,00	0,03	9,30E-01	1,00E+00
SHPS1	0,00	0,03	9,31E-01	1,00E+00
AIFM1	0,00	0,01	9,32E-01	1,00E+00
SCGB3A2	0,00	0,01	9,43E-01	1,00E+00
CD163	0,00	0,02	9,47E-01	1,00E+00
BANK1	0,00	0,01	9,53E-01	1,00E+00
IL6RA	0,00	0,03	9,53E-01	1,00E+00
MAP4K5	0,00	0,01	9,53E-01	1,00E+00
THPO	0,00	0,03	9,55E-01	1,00E+00
DECR1	0,00	0,01	9,60E-01	1,00E+00
CA5A	0,00	0,01	9,61E-01	1,00E+00
ST2	0,00	0,02	9,62E-01	1,00E+00
AZU1	0,00	0,02	9,73E-01	1,00E+00
HO1	0,00	0,03	9,74E-01	1,00E+00
NEMO	0,00	0,01	9,74E-01	1,00E+00
CXCL6	0,00	0,01	9,76E-01	1,00E+00
CCL11	0,00	0,03	9,76E-01	1,00E+00
ENAH	0,00	0,02	9,79E-01	1,00E+00
CA1	0,00	0,02	9,81E-01	1,00E+00
PLIN1	0,00	0,02	9,82E-01	1,00E+00
PAI	0,00	0,02	9,83E-01	1,00E+00
PRSS2	0,00	0,02	9,83E-01	1,00E+00
PCSK9	0,00	0,03	9,86E-01	1,00E+00
RRM2B	0,00	0,02	9,87E-01	1,00E+00
PVALB	0,00	0,01	9,89E-01	1,00E+00
PRELP	0,00	0,06	9,90E-01	1,00E+00
IL20RA	0,00	0,03	9,96E-01	1,00E+00
IL1RL2	0,00	0,02	9,97E-01	1,00E+00

Supplemental Table 6: Associations between proteins levels measured at V4 and ΔeGFR≥15mL/min/1.7m².

Multivariable logistic regression models included including the following variables at V1: age, sex, body mass index, current smoker and eGFR.

Protein at V4	β coefficient	Standard error	P value	Bonferroni P value
NPPC	0,12	0,02	2,92E-10	1,31E-07
MEPE	0,15	0,02	3,53E-10	1,58E-07
PLC	0,21	0,04	6,04E-09	2,70E-06
TNFR1	0,18	0,03	8,01E-09	3,58E-06
KLK6	0,18	0,03	1,22E-07	5,45E-05
EPHB4	0,20	0,04	1,64E-07	7,33E-05
TNFR2	0,15	0,03	2,41E-07	1,08E-04
LTBR	0,17	0,03	5,38E-07	2,40E-04
TNFRSF9	0,12	0,03	2,86E-06	1,28E-03
CLEC1A	0,15	0,03	3,76E-06	1,68E-03
CA14	0,16	0,04	6,78E-06	3,03E-03
TNFRSF11A	0,12	0,03	1,13E-05	5,05E-03
PGF	0,17	0,04	1,38E-05	6,17E-03
TFF3	0,11	0,02	1,51E-05	6,75E-03
MB	0,09	0,02	1,62E-05	7,24E-03
PTK7	0,14	0,03	3,13E-05	1,40E-02
IGFBP6	0,11	0,03	3,99E-05	1,78E-02
PI3	0,12	0,03	8,12E-05	3,63E-02
FABP4	0,07	0,02	8,21E-05	3,67E-02
DLK1	0,07	0,02	9,00E-05	4,02E-02
CD4	0,12	0,03	9,08E-05	4,06E-02
TNFRSF13B	0,11	0,03	1,35E-04	6,05E-02
CST3	0,09	0,02	1,44E-04	6,44E-02
PDL1	0,08	0,02	1,45E-04	6,49E-02
FABP2	0,05	0,01	1,53E-04	6,82E-02
PGLYRP1	0,08	0,02	1,88E-04	8,40E-02
TGFalpha	0,14	0,04	1,90E-04	8,49E-02
OPN	0,09	0,02	2,39E-04	1,07E-01
FGF23	0,09	0,02	2,57E-04	1,15E-01
CCL15	0,08	0,02	2,90E-04	1,30E-01
AGRP	0,10	0,03	3,84E-04	1,72E-01
CD93	0,12	0,04	3,88E-04	1,73E-01
Gal9	0,12	0,03	3,93E-04	1,76E-01
ADM	0,09	0,03	6,65E-04	2,97E-01
BAMBI	0,10	0,03	8,82E-04	3,94E-01
CCL23	0,07	0,02	1,11E-03	4,96E-01

Gal4	0,07	0,02	1,16E-03	5,19E-01
PRSS2	0,06	0,02	1,21E-03	5,43E-01
TRAILR2	0,07	0,02	1,27E-03	5,70E-01
GDNF	0,07	0,02	1,89E-03	8,44E-01
MERTK	0,08	0,03	2,17E-03	9,72E-01
MMP7	0,08	0,03	2,23E-03	9,96E-01
CX3CL1	0,09	0,03	2,52E-03	1,00E+00
REG1A	0,05	0,02	2,88E-03	1,00E+00
GDF15	0,07	0,02	2,99E-03	1,00E+00
TNFRSF10A	0,09	0,03	3,01E-03	1,00E+00
PECAM1	-0,04	0,01	3,28E-03	1,00E+00
COL18A1	0,08	0,03	3,29E-03	1,00E+00
SCF	0,08	0,03	3,47E-03	1,00E+00
ALCAM	0,13	0,04	3,77E-03	1,00E+00
IL12B	0,05	0,02	4,17E-03	1,00E+00
Gal3	0,09	0,03	4,49E-03	1,00E+00
MMP3	0,05	0,02	5,41E-03	1,00E+00
COL1A1	0,07	0,03	6,91E-03	1,00E+00
RAGE	0,08	0,03	7,53E-03	1,00E+00
CAPG	0,05	0,02	8,00E-03	1,00E+00
CAPG	0,05	0,02	8,00E-03	1,00E+00
TM	0,08	0,03	8,59E-03	1,00E+00
REN	0,04	0,01	9,16E-03	1,00E+00
AMN	0,04	0,01	1,03E-02	1,00E+00
IL18BP	0,08	0,03	1,30E-02	1,00E+00
CD5	0,07	0,03	1,34E-02	1,00E+00
IL2RA	0,06	0,02	1,34E-02	1,00E+00
CCL14	0,05	0,02	1,34E-02	1,00E+00
THBS4	-0,05	0,02	1,40E-02	1,00E+00
TNFRSF10C	0,05	0,02	1,49E-02	1,00E+00
FABP9	0,06	0,03	1,52E-02	1,00E+00
SELP	-0,03	0,01	1,52E-02	1,00E+00
DSG4	0,05	0,02	1,66E-02	1,00E+00
PRSS27	0,05	0,02	1,75E-02	1,00E+00
IGFBP7	0,08	0,03	1,98E-02	1,00E+00
PDCD1	0,07	0,03	2,01E-02	1,00E+00
CSTB	0,04	0,02	2,03E-02	1,00E+00
RARRES2	0,07	0,03	2,10E-02	1,00E+00
COMP	-0,05	0,02	2,19E-02	1,00E+00
CPA1	0,03	0,01	2,30E-02	1,00E+00
GT	0,04	0,02	2,46E-02	1,00E+00
NTproBNP	0,03	0,01	2,72E-02	1,00E+00
CPB1	0,03	0,02	2,78E-02	1,00E+00
LIF	0,06	0,03	2,82E-02	1,00E+00
FCN2	-0,04	0,02	2,93E-02	1,00E+00
AXL	0,07	0,03	2,96E-02	1,00E+00

CA12	0,08	0,04	3,43E-02	1,00E+00
CASP3	-0,02	0,01	3,54E-02	1,00E+00
IGLC2	0,04	0,02	3,56E-02	1,00E+00
LRP1	-0,05	0,02	3,72E-02	1,00E+00
TLT2	0,05	0,02	3,87E-02	1,00E+00
RETN	0,04	0,02	4,00E-02	1,00E+00
MCP3	0,04	0,02	4,14E-02	1,00E+00
JAMA	-0,02	0,01	4,32E-02	1,00E+00
MMP10	0,04	0,02	4,39E-02	1,00E+00
SPON2	0,10	0,05	4,45E-02	1,00E+00
C2	-0,05	0,03	4,47E-02	1,00E+00
IL33	-0,29	0,13	4,48E-02	1,00E+00
LHB	0,02	0,01	4,53E-02	1,00E+00
DCN	0,09	0,04	4,58E-02	1,00E+00
CTSL1	0,06	0,03	4,59E-02	1,00E+00
CD59	0,06	0,03	4,93E-02	1,00E+00
CSF1	0,10	0,05	5,02E-02	1,00E+00
IL10RB	0,07	0,04	5,28E-02	1,00E+00
Notch3	0,06	0,03	5,55E-02	1,00E+00
TF	0,07	0,03	5,68E-02	1,00E+00
TNFRSF14	0,05	0,02	5,89E-02	1,00E+00
TGFBR3	0,05	0,03	5,96E-02	1,00E+00
PLIN1	0,04	0,02	6,03E-02	1,00E+00
MBL2	0,01	0,01	6,13E-02	1,00E+00
CCL16	0,04	0,02	6,38E-02	1,00E+00
UPAR	0,05	0,02	6,54E-02	1,00E+00
SERPINA9	0,03	0,02	6,64E-02	1,00E+00
SERPINA7	-0,05	0,03	6,79E-02	1,00E+00
LDLreceptor	-0,03	0,02	7,28E-02	1,00E+00
CALCA	0,03	0,02	7,37E-02	1,00E+00
hOSCAR	0,07	0,04	7,39E-02	1,00E+00
IL17C	0,03	0,02	7,58E-02	1,00E+00
ARTN	0,04	0,02	7,82E-02	1,00E+00
PON2	-0,04	0,02	7,87E-02	1,00E+00
VASN	0,06	0,03	8,01E-02	1,00E+00
TRAP	-0,05	0,03	8,40E-02	1,00E+00
IL4RA	0,06	0,04	8,50E-02	1,00E+00
TIMP4	0,04	0,03	8,52E-02	1,00E+00
GLO1	-0,02	0,01	8,54E-02	1,00E+00
CALR	-0,07	0,04	8,63E-02	1,00E+00
TIE1	-0,07	0,04	8,95E-02	1,00E+00
PLTP	-0,05	0,03	8,97E-02	1,00E+00
CHL1	-0,05	0,03	8,99E-02	1,00E+00
OSMR	-0,07	0,04	9,18E-02	1,00E+00
IL18	-0,03	0,02	9,29E-02	1,00E+00
ANG	0,03	0,02	9,40E-02	1,00E+00

MAEA	-0,04	0,03	9,47E-02	1,00E+00
FGR	-0,02	0,01	9,72E-02	1,00E+00
tPA	-0,02	0,01	1,02E-01	1,00E+00
CNTN1	0,06	0,04	1,06E-01	1,00E+00
PTX3	0,04	0,02	1,07E-01	1,00E+00
YES1	-0,01	0,01	1,08E-01	1,00E+00
CA4	0,04	0,02	1,09E-01	1,00E+00
CRTAC1	-0,03	0,02	1,10E-01	1,00E+00
APOM	-0,04	0,03	1,11E-01	1,00E+00
IGFBP2	0,03	0,02	1,11E-01	1,00E+00
NEMO	-0,01	0,01	1,12E-01	1,00E+00
AMBP	0,08	0,05	1,14E-01	1,00E+00
IL1RT1	0,06	0,04	1,15E-01	1,00E+00
TGM2	0,03	0,02	1,16E-01	1,00E+00
PIgR	0,12	0,07	1,17E-01	1,00E+00
ST1A1	-0,02	0,01	1,18E-01	1,00E+00
CST5	0,03	0,02	1,18E-01	1,00E+00
WAS	-0,02	0,01	1,19E-01	1,00E+00
AGR2	0,02	0,01	1,23E-01	1,00E+00
SPON1	0,07	0,05	1,27E-01	1,00E+00
PCOLCE	0,03	0,02	1,27E-01	1,00E+00
STAMBP	-0,02	0,01	1,29E-01	1,00E+00
CTSZ	0,04	0,03	1,30E-01	1,00E+00
BANK1	-0,01	0,01	1,31E-01	1,00E+00
MAP4K5	-0,01	0,01	1,38E-01	1,00E+00
GRN	0,05	0,03	1,38E-01	1,00E+00
PRELP	0,08	0,05	1,40E-01	1,00E+00
FETUB	-0,04	0,02	1,42E-01	1,00E+00
CCL19	0,01	0,01	1,44E-01	1,00E+00
CTSD	-0,03	0,02	1,48E-01	1,00E+00
MFAP5	0,05	0,03	1,49E-01	1,00E+00
VSIG2	0,03	0,02	1,51E-01	1,00E+00
DECR1	-0,01	0,01	1,52E-01	1,00E+00
BNP	0,04	0,03	1,57E-01	1,00E+00
MCP1	0,03	0,02	1,57E-01	1,00E+00
SIRT5	0,05	0,03	1,63E-01	1,00E+00
LTBP2	-0,26	0,18	1,64E-01	1,00E+00
TR	0,03	0,02	1,66E-01	1,00E+00
SRC	-0,02	0,01	1,68E-01	1,00E+00
CA5A	0,02	0,01	1,71E-01	1,00E+00
TCN2	-0,03	0,02	1,78E-01	1,00E+00
SHPS1	0,03	0,03	1,79E-01	1,00E+00
uPA	-0,03	0,02	1,80E-01	1,00E+00
TIMP1	0,03	0,02	1,82E-01	1,00E+00
IL13	-0,03	0,02	1,84E-01	1,00E+00
CCL4	-0,02	0,02	1,86E-01	1,00E+00

NT3	0,03	0,02	1,88E-01	1,00E+00
SAA4	-0,02	0,02	1,88E-01	1,00E+00
RASSF2	-0,02	0,02	1,90E-01	1,00E+00
IDUA	-0,03	0,02	1,93E-01	1,00E+00
LTA4H	-0,41	0,22	1,96E-01	1,00E+00
IL18	-0,02	0,02	1,97E-01	1,00E+00
METAP1	0,14	0,11	1,97E-01	1,00E+00
IL6RA	0,03	0,03	2,01E-01	1,00E+00
IL1ra	0,02	0,02	2,02E-01	1,00E+00
ALDH3A1	0,02	0,02	2,03E-01	1,00E+00
IL16	0,02	0,02	2,03E-01	1,00E+00
F7	-0,03	0,03	2,05E-01	1,00E+00
UM	-0,06	0,04	2,06E-01	1,00E+00
TNFSF13B	0,04	0,03	2,07E-01	1,00E+00
IL1RT2	0,04	0,03	2,07E-01	1,00E+00
FGF5	0,09	0,07	2,07E-01	1,00E+00
IL7	0,02	0,01	2,07E-01	1,00E+00
CLSPN	-0,07	0,06	2,09E-01	1,00E+00
ANG1	0,01	0,01	2,13E-01	1,00E+00
PROC	-0,03	0,02	2,14E-01	1,00E+00
RARRES1	-0,05	0,04	2,21E-01	1,00E+00
STX8	-0,02	0,01	2,24E-01	1,00E+00
ANGPTL3	-0,03	0,03	2,25E-01	1,00E+00
VEGFA	0,03	0,03	2,25E-01	1,00E+00
EGFL7	0,03	0,02	2,29E-01	1,00E+00
PTN	0,02	0,02	2,31E-01	1,00E+00
VCAM1	0,03	0,03	2,35E-01	1,00E+00
STK4	-0,01	0,01	2,35E-01	1,00E+00
S2	0,05	0,04	2,39E-01	1,00E+00
PON3	-0,02	0,02	2,43E-01	1,00E+00
TNC	-0,02	0,02	2,45E-01	1,00E+00
XCL1	0,02	0,02	2,47E-01	1,00E+00
ITGB2	-0,02	0,02	2,51E-01	1,00E+00
MMP12	0,02	0,02	2,52E-01	1,00E+00
ITGAM	-0,04	0,04	2,54E-01	1,00E+00
SCGB3A2	0,01	0,01	2,58E-01	1,00E+00
DNER	-0,05	0,05	2,60E-01	1,00E+00
PAPPA	0,02	0,02	2,64E-01	1,00E+00
RCOR1	-0,03	0,03	2,65E-01	1,00E+00
MAX	-0,02	0,02	2,65E-01	1,00E+00
HSP27	-0,02	0,02	2,66E-01	1,00E+00
SMAD1	-0,02	0,01	2,69E-01	1,00E+00
CNDP1	-0,03	0,02	2,73E-01	1,00E+00
TFPI	-0,03	0,03	2,74E-01	1,00E+00
F11	-0,03	0,03	2,77E-01	1,00E+00
AXIN1	-0,01	0,01	2,79E-01	1,00E+00

CNTN2	-0,02	0,02	2,81E-01	1,00E+00
ITGB1BP1	-0,07	0,07	2,84E-01	1,00E+00
ITGB1BP2	-0,01	0,01	2,84E-01	1,00E+00
CA3	0,03	0,03	2,84E-01	1,00E+00
BMP6	0,03	0,02	2,90E-01	1,00E+00
GDF2	-0,03	0,03	2,95E-01	1,00E+00
GAS6	-0,03	0,03	2,97E-01	1,00E+00
TOP2B	-0,01	0,01	3,00E-01	1,00E+00
FCGR2A	-0,02	0,02	3,02E-01	1,00E+00
NUCB2	0,02	0,02	3,06E-01	1,00E+00
MVK	-0,01	0,01	3,06E-01	1,00E+00
BID	-0,03	0,03	3,10E-01	1,00E+00
FAP	-0,03	0,03	3,13E-01	1,00E+00
SELE	-0,02	0,02	3,14E-01	1,00E+00
PTPRS	0,06	0,06	3,22E-01	1,00E+00
CXCL9	0,01	0,01	3,24E-01	1,00E+00
EGFR	0,05	0,05	3,29E-01	1,00E+00
VEGFC	0,02	0,02	3,33E-01	1,00E+00
PRKAB1	-0,01	0,01	3,36E-01	1,00E+00
TNFB	0,02	0,02	3,37E-01	1,00E+00
CXCL16	0,04	0,04	3,39E-01	1,00E+00
IL27	0,02	0,03	3,40E-01	1,00E+00
Dkk1	0,01	0,02	3,42E-01	1,00E+00
PVALB	-0,01	0,01	3,46E-01	1,00E+00
MEGF9	-0,03	0,03	3,48E-01	1,00E+00
CD8A	0,01	0,02	3,63E-01	1,00E+00
MMP9	0,01	0,01	3,67E-01	1,00E+00
SERPINA5	-0,02	0,02	3,68E-01	1,00E+00
TNNI3	0,01	0,01	3,70E-01	1,00E+00
HGF	-0,02	0,03	3,72E-01	1,00E+00
PRKRA	-0,01	0,02	3,75E-01	1,00E+00
CCL11	0,02	0,02	3,75E-01	1,00E+00
vWF	0,01	0,01	3,76E-01	1,00E+00
GP6	-0,01	0,01	3,82E-01	1,00E+00
BOC	-0,03	0,04	3,83E-01	1,00E+00
C1QTNF1	-0,01	0,02	3,84E-01	1,00E+00
INPPL1	-0,01	0,01	3,86E-01	1,00E+00
LYVE1	-0,02	0,03	3,87E-01	1,00E+00
AZU1	-0,01	0,01	3,88E-01	1,00E+00
BetaNGF	0,02	0,03	3,88E-01	1,00E+00
LILRB5	0,01	0,01	3,97E-01	1,00E+00
ICAM3	-0,02	0,02	3,97E-01	1,00E+00
LPL	0,02	0,02	4,04E-01	1,00E+00
CXCL10	0,01	0,01	4,10E-01	1,00E+00
IGFBP1	0,01	0,01	4,11E-01	1,00E+00
CR2	0,02	0,02	4,12E-01	1,00E+00

SLAMF1	0,02	0,02	4,22E-01	1,00E+00
CRH	0,01	0,01	4,25E-01	1,00E+00
TGFBI	0,02	0,02	4,33E-01	1,00E+00
AIFM1	-0,01	0,01	4,34E-01	1,00E+00
CDH5	0,03	0,03	4,36E-01	1,00E+00
PARP1	0,01	0,01	4,41E-01	1,00E+00
IL20RA	0,02	0,02	4,43E-01	1,00E+00
SIRT2	-0,01	0,01	4,52E-01	1,00E+00
CES1	-0,01	0,01	4,58E-01	1,00E+00
ATP6AP2	-0,03	0,04	4,61E-01	1,00E+00
GH	0,00	0,01	4,62E-01	1,00E+00
AOC3	-0,02	0,03	4,64E-01	1,00E+00
EDIL3	0,04	0,06	4,70E-01	1,00E+00
CTRC	0,01	0,01	4,73E-01	1,00E+00
THBS2	0,03	0,05	4,73E-01	1,00E+00
IL1RL2	-0,02	0,02	4,77E-01	1,00E+00
PPM1B	-0,07	0,10	4,77E-01	1,00E+00
PLXNB2	-0,03	0,04	4,80E-01	1,00E+00
ICAM1	-0,02	0,03	4,82E-01	1,00E+00
MCP4	0,01	0,01	4,84E-01	1,00E+00
ENTPD6	-0,03	0,04	4,88E-01	1,00E+00
PDL2	0,02	0,03	4,89E-01	1,00E+00
CCL25	0,01	0,02	4,93E-01	1,00E+00
ENG	-0,02	0,03	4,95E-01	1,00E+00
LAT2	-0,01	0,01	4,98E-01	1,00E+00
SERPINA12	0,01	0,01	5,00E-01	1,00E+00
FAS	0,02	0,03	5,00E-01	1,00E+00
RASA1	0,02	0,02	5,01E-01	1,00E+00
PTPRJ	-0,01	0,02	5,03E-01	1,00E+00
LIFR	0,03	0,04	5,13E-01	1,00E+00
IL2RB	-0,02	0,04	5,17E-01	1,00E+00
CEACAM8	0,01	0,02	5,17E-01	1,00E+00
IL8	0,01	0,01	5,18E-01	1,00E+00
PRCP	0,05	0,07	5,20E-01	1,00E+00
LEP	0,01	0,01	5,24E-01	1,00E+00
CHIT1	0,01	0,01	5,34E-01	1,00E+00
TSLP	-0,04	0,07	5,35E-01	1,00E+00
ERBB2IP	-0,01	0,02	5,36E-01	1,00E+00
PLXDC1	-0,02	0,04	5,43E-01	1,00E+00
FOXO1	0,01	0,01	5,44E-01	1,00E+00
CD40L	-0,01	0,01	5,44E-01	1,00E+00
TMPRSS15	-0,02	0,03	5,46E-01	1,00E+00
NUB1	-0,01	0,02	5,48E-01	1,00E+00
DPP4	-0,02	0,03	5,48E-01	1,00E+00
PDGFsubunitB	0,01	0,01	5,50E-01	1,00E+00
KIM1	0,01	0,01	5,55E-01	1,00E+00

TNF	-0,01	0,03	5,55E-01	1,00E+00
NRP1	0,03	0,05	5,55E-01	1,00E+00
EpCAM	0,01	0,01	5,59E-01	1,00E+00
IL18R1	0,01	0,03	5,63E-01	1,00E+00
PRSS8	0,02	0,03	5,66E-01	1,00E+00
PSPD	0,01	0,01	5,71E-01	1,00E+00
GALNT10	0,02	0,04	5,73E-01	1,00E+00
IL10	-0,01	0,02	5,74E-01	1,00E+00
CFHR5	-0,01	0,02	5,74E-01	1,00E+00
CCL18	0,01	0,01	5,75E-01	1,00E+00
NCF2	-0,01	0,01	5,78E-01	1,00E+00
CD163	-0,01	0,02	5,89E-01	1,00E+00
TNFSF14	-0,01	0,02	5,97E-01	1,00E+00
ADAMTS13	-0,03	0,06	6,08E-01	1,00E+00
IL4	0,01	0,02	6,09E-01	1,00E+00
APN	0,02	0,04	6,12E-01	1,00E+00
EPO	-0,01	0,01	6,13E-01	1,00E+00
Flt3L	-0,01	0,03	6,23E-01	1,00E+00
PSMA1	-0,01	0,01	6,25E-01	1,00E+00
THPO	0,01	0,03	6,29E-01	1,00E+00
NRTN	-0,02	0,04	6,30E-01	1,00E+00
CSNK1D	-0,01	0,03	6,32E-01	1,00E+00
LILRB2	-0,01	0,02	6,35E-01	1,00E+00
SELL	-0,01	0,03	6,43E-01	1,00E+00
TRANCE	0,01	0,02	6,49E-01	1,00E+00
SLAMF7	-0,01	0,02	6,55E-01	1,00E+00
IFNgamma	0,14	0,30	6,58E-01	1,00E+00
ENTPD2	-0,02	0,05	6,59E-01	1,00E+00
TWEAK	0,02	0,04	6,61E-01	1,00E+00
MMP2	0,01	0,03	6,62E-01	1,00E+00
CCL28	0,01	0,02	6,62E-01	1,00E+00
CXCL11	0,00	0,01	6,63E-01	1,00E+00
LOX1	-0,01	0,02	6,65E-01	1,00E+00
IgGFcreceptorIIb	-0,01	0,01	6,65E-01	1,00E+00
IL17RA	0,01	0,02	6,68E-01	1,00E+00
OSM	0,00	0,01	6,70E-01	1,00E+00
QPCT	-0,01	0,03	6,73E-01	1,00E+00
MCP2	0,01	0,02	6,84E-01	1,00E+00
PAI	-0,01	0,01	6,86E-01	1,00E+00
CXCL1	0,00	0,01	6,89E-01	1,00E+00
IL17D	0,01	0,04	6,92E-01	1,00E+00
ACE2	-0,01	0,02	6,93E-01	1,00E+00
CCL17	0,00	0,01	7,00E-01	1,00E+00
CD46	0,01	0,02	7,05E-01	1,00E+00
ST3GAL1	0,01	0,02	7,06E-01	1,00E+00
DPP6	0,01	0,03	7,10E-01	1,00E+00

MET	-0,01	0,03	7,12E-01	1,00E+00
NBN	-0,01	0,01	7,12E-01	1,00E+00
CD40	0,01	0,02	7,16E-01	1,00E+00
TIMD4	-0,01	0,02	7,17E-01	1,00E+00
CDCP1	-0,01	0,02	7,17E-01	1,00E+00
VASH1	-0,01	0,03	7,18E-01	1,00E+00
CDH1	-0,01	0,02	7,20E-01	1,00E+00
MMP1	0,00	0,01	7,23E-01	1,00E+00
TIE2	0,01	0,04	7,24E-01	1,00E+00
ENRAGE	0,01	0,02	7,25E-01	1,00E+00
NID1	0,01	0,02	7,25E-01	1,00E+00
HPGDS	0,01	0,02	7,28E-01	1,00E+00
ICAM2	0,01	0,03	7,33E-01	1,00E+00
LAPTGFbeta1	-0,01	0,03	7,34E-01	1,00E+00
HBEGF	0,00	0,01	7,36E-01	1,00E+00
CXCL6	0,00	0,01	7,37E-01	1,00E+00
FGF21	0,00	0,01	7,41E-01	1,00E+00
IL22RA1	-0,04	0,12	7,46E-01	1,00E+00
LILRB1	0,01	0,03	7,56E-01	1,00E+00
GIF	0,00	0,01	7,56E-01	1,00E+00
IL20	-0,01	0,03	7,60E-01	1,00E+00
IL6	0,00	0,01	7,62E-01	1,00E+00
CCL5	0,00	0,01	7,62E-01	1,00E+00
BTC	0,00	0,01	7,63E-01	1,00E+00
LCN2	-0,02	0,07	7,64E-01	1,00E+00
ADGRG1	0,00	0,01	7,73E-01	1,00E+00
PXN	0,00	0,02	7,75E-01	1,00E+00
CCL20	0,00	0,01	7,82E-01	1,00E+00
PSGL1	-0,01	0,04	7,83E-01	1,00E+00
VEGFD	-0,01	0,03	7,85E-01	1,00E+00
PDGFC	-0,01	0,04	7,87E-01	1,00E+00
EFEMP1	0,01	0,03	7,90E-01	1,00E+00
CHI3L1	0,00	0,01	7,99E-01	1,00E+00
ADA	0,01	0,03	8,00E-01	1,00E+00
RRM2B	0,00	0,02	8,02E-01	1,00E+00
CA1	0,00	0,01	8,05E-01	1,00E+00
FES	0,01	0,03	8,08E-01	1,00E+00
IL15RA	0,04	0,16	8,11E-01	1,00E+00
PDGFsubunitA	0,00	0,01	8,17E-01	1,00E+00
GP1BA	0,00	0,01	8,22E-01	1,00E+00
IL17A	0,00	0,02	8,23E-01	1,00E+00
TNXB	0,01	0,05	8,24E-01	1,00E+00
PAM	-0,01	0,03	8,26E-01	1,00E+00
ST6GAL1	0,00	0,02	8,34E-01	1,00E+00
BLMhydrolase	0,00	0,02	8,46E-01	1,00E+00
CASP8	0,00	0,02	8,63E-01	1,00E+00

HO1	0,00	0,03	8,65E-01	1,00E+00
CES2	-0,01	0,04	8,67E-01	1,00E+00
NOS3	0,00	0,03	8,67E-01	1,00E+00
DEFA1	-0,01	0,04	8,79E-01	1,00E+00
E4BP1	0,00	0,01	8,79E-01	1,00E+00
IL1alpha	0,02	0,14	8,80E-01	1,00E+00
GNLY	0,00	0,02	8,83E-01	1,00E+00
HAOX1	0,00	0,01	8,85E-01	1,00E+00
SORT1	0,00	0,03	8,86E-01	1,00E+00
MPO	0,00	0,02	8,90E-01	1,00E+00
PLA2G7	-0,01	0,04	8,95E-01	1,00E+00
ST2	0,00	0,02	8,98E-01	1,00E+00
IL5	0,00	0,01	8,99E-01	1,00E+00
FOSB	0,00	0,02	8,99E-01	1,00E+00
FGF19	0,00	0,01	9,03E-01	1,00E+00
NCAM1	0,00	0,03	9,04E-01	1,00E+00
CCL24	0,00	0,01	9,05E-01	1,00E+00
TIGAR	0,00	0,02	9,09E-01	1,00E+00
PAR1	0,00	0,02	9,18E-01	1,00E+00
PCSK9	0,00	0,03	9,21E-01	1,00E+00
CXCL5	0,00	0,01	9,22E-01	1,00E+00
IL10RA	0,00	0,02	9,22E-01	1,00E+00
IGFBP3	0,00	0,03	9,33E-01	1,00E+00
MARCO	0,00	0,05	9,35E-01	1,00E+00
TRAIL	0,00	0,04	9,36E-01	1,00E+00
FS	0,00	0,02	9,37E-01	1,00E+00
SPARCL1	0,00	0,03	9,42E-01	1,00E+00
CD244	0,00	0,03	9,42E-01	1,00E+00
FCGR3B	0,00	0,02	9,42E-01	1,00E+00
OPG	0,00	0,03	9,50E-01	1,00E+00
IL24	0,00	0,03	9,54E-01	1,00E+00
CD84	0,00	0,02	9,57E-01	1,00E+00
MAGED1	0,00	0,03	9,61E-01	1,00E+00
CD6	0,00	0,02	9,63E-01	1,00E+00
PDP1	0,01	0,16	9,64E-01	1,00E+00
FKBP1B	0,00	0,02	9,69E-01	1,00E+00
ENAH	0,00	0,03	9,78E-01	1,00E+00
S1	0,00	0,07	9,83E-01	1,00E+00
KIT	0,00	0,03	9,85E-01	1,00E+00
STXBP3	0,00	0,03	9,89E-01	1,00E+00
IL7R	0,00	0,02	9,90E-01	1,00E+00
NOTCH1	0,00	0,04	9,92E-01	1,00E+00
PRTN3	0,00	0,01	9,97E-01	1,00E+00

Supplemental Table 7: Associations between genes expressions measured at V4 and ΔeGFR≥15mL/min/1.7m^2.

Multivariable logistic regression models included including the following variables at V1: age, sex, body mass index, current smoker and eGFR.

Gene at V4	β coefficient	Standard error	P value	Bonferroni P value
LOC102724434_AC073130.1	0,14	0,03	1,02E-06	3,47E-05
CCL18	0,16	0,03	9,44E-06	3,21E-04
RPL36AP51	-0,08	0,02	1,52E-05	5,17E-04
RPL21P10	-0,09	0,02	1,93E-05	6,56E-04
SESN3	-0,06	0,01	2,49E-05	8,47E-04
OR5M4P	0,10	0,02	2,96E-05	1,01E-03
LYRM5	-0,08	0,02	3,07E-05	1,04E-03
LINC00681	-0,11	0,03	3,22E-05	1,09E-03
NEIL3	-0,09	0,02	7,38E-05	2,51E-03
MIR1205	0,07	0,02	7,91E-05	2,69E-03
RP11_266O8.1	0,15	0,04	1,65E-04	5,78E-03
DNAJC6	-0,04	0,01	2,09E-04	7,14E-03
CYP46A1	0,11	0,03	2,52E-04	8,50E-03
RP11_494K3.2	-0,12	0,03	3,80E-04	1,29E-02
RP11_736K20.4_RP11_736K20.5	-0,11	0,03	4,24E-04	1,43E-02
CXADRP2	0,10	0,03	6,23E-04	2,11E-02
VEGFC	0,06	0,02	9,95E-03	3,38E-01
THBS1	-0,02	0,01	6,64E-02	1,00E+00
KLK6	0,08	0,04	7,53E-02	1,00E+00
VEGFB	0,04	0,03	1,83E-01	1,00E+00
MMP2	0,04	0,03	1,90E-01	1,00E+00
PIGF	-0,02	0,02	2,53E-01	1,00E+00
F3	-0,04	0,04	2,81E-01	1,00E+00
VCAM1	-0,03	0,03	3,12E-01	1,00E+00
FLT1	0,03	0,03	3,47E-01	1,00E+00
VEGFA	0,02	0,02	3,74E-01	1,00E+00
LYRM5.1	-0,01	0,02	4,11E-01	1,00E+00
TGFA	-0,01	0,02	5,49E-01	1,00E+00
ICAM1	0,01	0,03	6,37E-01	1,00E+00
TIMP4	-0,01	0,03	7,25E-01	1,00E+00
TIMP1	-0,01	0,01	7,29E-01	1,00E+00
FGF23	0,01	0,05	8,27E-01	1,00E+00
SESN3.1	0,00	0,01	8,49E-01	1,00E+00
PGF	0,01	0,04	8,93E-01	1,00E+00

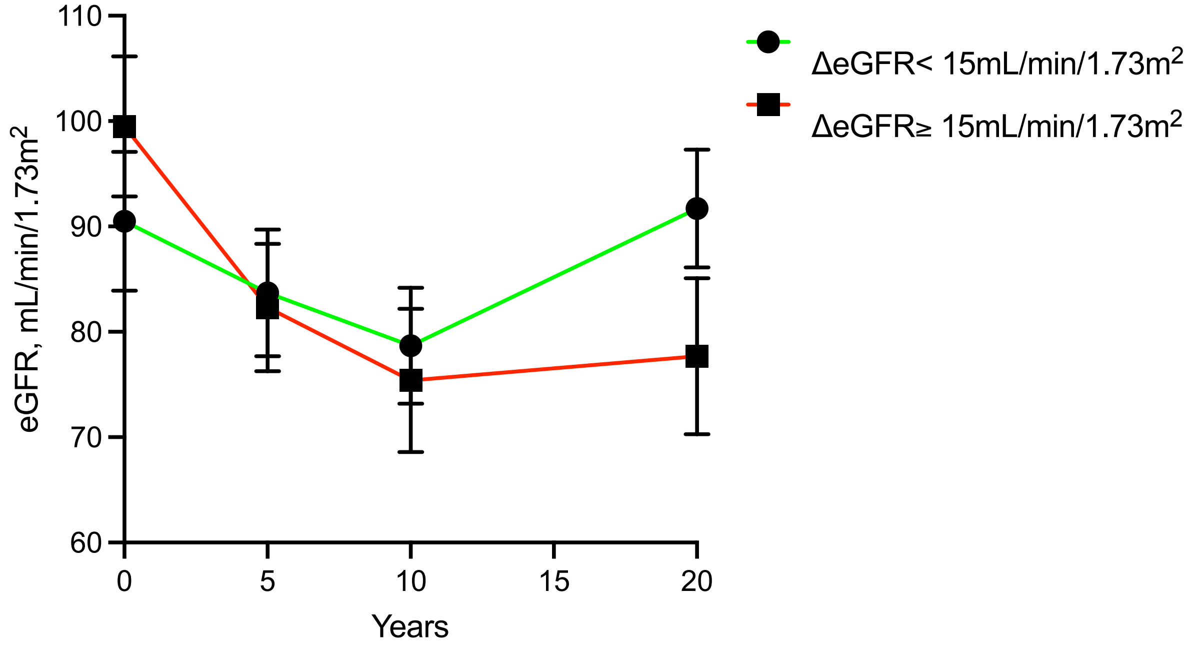

Supplemental Figure 1: Kidney function decline over 20 years

Estimated glomerular filtration rate (eGFR) decrease over 20 years in subjects who experienced ΔeGFR≥15mL/min/1.7m^2 (red line) and those who did not (green line). Baseline corresponds to visit 1 (V1, n= 1087), year 5 to V2 (n= 895), year 10 to V3 (n= 540) and year 20 to V4 (n= 1087).

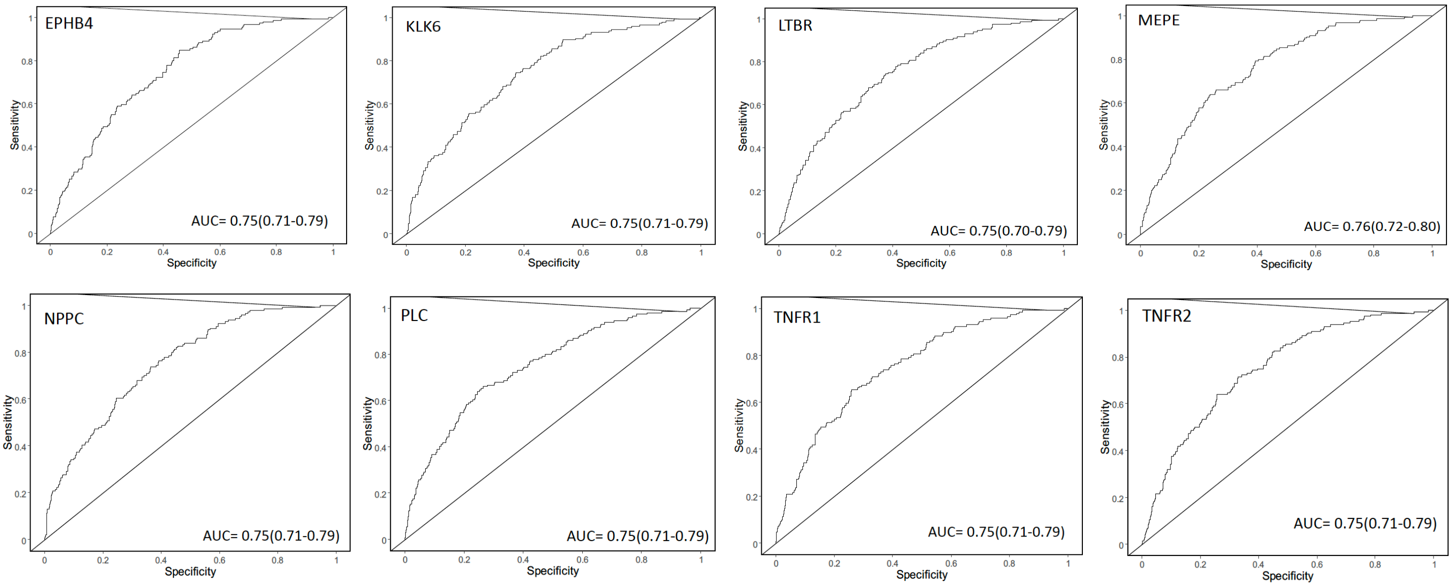

Supplemental Figure 2: Predictive ability of proteins levels measured at V4 for new-onset kidney function decline.

The predictive ability of the top proteins levels measured at V4 for ΔeGFR≥15mL/min/1.7m^2 was studied by calculating receiver operating characteristic (ROC) curves with their corresponding area under the curve (AUC) and 95% confidence interval.

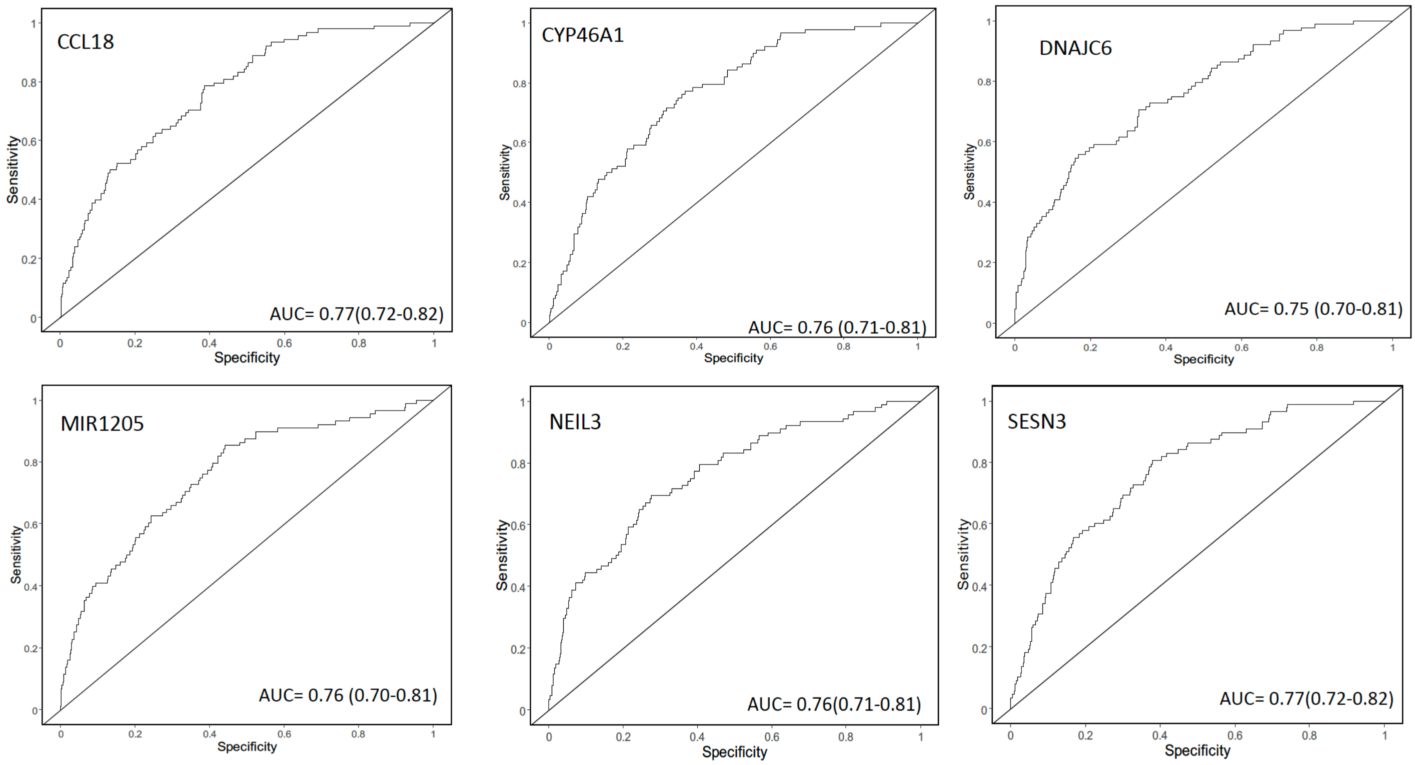

Supplemental Figure 3: Predictive ability of genes expressions measured at V4 for new-onset kidney function decline.

The predictive ability of the top genes esxpressions measured at V4 for ΔeGFR≥15mL/min/1.7m^2 was studied by calculating receiver operating characteristic (ROC) curves with their corresponding area under the curve (AUC) and 95% confidence interval.
